# FN-silk membrane enables alveologenesis processes and self-organization of the H441 epithelial cell line into native-like alveolar morphology

**DOI:** 10.1038/s41598-026-59951-4

**Published:** 2026-06-27

**Authors:** Savvini Gkouma, Linnea Påvenius, Linnea Gustafsson, Christos Tasiopoulos, André Charbonneau, Swapna Upadhyay, Hjalmar Brismar, Lena Palmberg, My Hedhammar

**Affiliations:** 1 Division of Protein Technology, Department of Protein Science, KTH Royal Institute of Technology, Stockholm, Sweden; 2 Science for Life Laboratory, Department Women’s and Children’s Health, Karolinska Institute, Stockholm, Sweden; 3 Division of Micro and Nanosystems, Department of Intelligent Systems, School of Electrical Engineering and Computer Science, KTH Royal Institute of Technology, Stockholm, Sweden; 4 Spiber Technologies AB, Stockholm, Sweden; 5 Institute of Environmental Medicine, Integrative Toxicology, Karolinska Institute, Stockholm, Sweden; 6 Science for Life Laboratory, Department of Applied Physics, KTH Royal Institute of Technology, Stockholm, Sweden; 7 Atlas Antibodies, Stockholm, Sweden

**Keywords:** Biological techniques, Biotechnology, Cell biology

## Abstract

In vitro alveolar-capillary models based on co-culturing the alveolar epithelial cell line H441 and primary pulmonary microvascular endothelial cells (HPMEC) are a widely used platform for evaluating the function of the alveolar barrier. However, the relatively thick synthetic membranes that are used as substrates in most approaches fail to mimic the properties of the natural basement membrane, thereby decreasing the physiological relevance of those models. We investigated the potential of the FN-silk membrane to support an in vitro alveolar-capillary model. The FN-silk membrane is micrometer-thin, fibrillar, constructed from a functionalized recombinant spider silk protein, and has been previously shown to be a potent basement membrane mimic supporting physiologically relevant in vitro models of various barrier tissues (i.e., blood vessel, skin, BBB, and kidney). Herein, it supported alveolar epithelial and endothelial barrier formation, surfactant protein B and C (SPB, SPC) production, and epithelial cell polarization, detected with immunofluorescence. Notably, we also demonstrated for the first time, to our knowledge, that key events related to alveologenesis (i.e., cell hollowing, lumen formation, septation, and *α*-SMA expression) can take place in an in vitro model. This further highlights the ability of the FN-silk membrane to recapitulate the complicated alveolar milieu and expanding the known potential of the H441 cell line, which to our knowledge, has not been previously reported to enable modeling the alveolar tissue 3D morphology. We propose the FN-silk-based alveolar-capillary model as an advanced in vitro model that can be used as a potent tool in respiratory, developmental biology, and regenerative medicine research.

**Supplementary Information:**

The online version contains supplementary material available at 10.1038/s41598-026-59951-4.

## Introduction

The most important function of the lung is gas exchange, which takes place in specialized air sacs located at the distal site of the respiratory tree—the alveoli. They are the functional units of lung tissue, constituting about 90% of its total volume, and are arranged in acini (sac-like cavities) located in the lung parenchyma^1–3^. Alveoli start developing postnatally at the alveolarization stage of lung development and drastically increase in numbers within the first eight years of life^1,2,4^. At the beginning of the alveolarization stage, the respiratory tree ends in saccules (i.e., wide luminal spaces), which mature into alveoli through events of secondary septation, cell hollowing, and ECM remodeling. During this alveologenesis process, the cell-cell and cell-ECM interactions of epithelial, endothelial cells and myofibroblasts are considered critical, however the exact mechanism driving it is yet to be fully understood^5–7^. The alveolar functional unit consists of two types of epithelial cells (type I and II), macrophages, vascular, and mesenchymal cells^1–4,8^. Although they originate from common alveolar progenitor cells, the two types of epithelial cells differ in number, morphology, and function. Alveolar type I cells (ATI) are highly flattened squamous epithelial cells that cover approximately 96% of the total alveolar surface area^1–4,8,9^, which facilitates their primary function: gas exchange with the underlying endothelium. Type II alveolar cells (ATII) are smaller but more abundant, have a cuboidal shape, and produce surfactants (e.g., surfactant protein B and C (SPB, SPC))—a mixture of phospholipids, proteins, and ions^3,9^, which reduce surface tension during breathing. They have a propensity for self-renewal, but also for differentiation into ATI cells in response to innate immune stimuli or injury^1–4,8–10^.

The alveolar wall is lined with both ATI and ATII cells and is extremely thin. It is separated from the adjacent capillary plexus by the respiratory membrane, a multilayered ultra-thin (0.1–1.5 µm) basement membrane rich in laminins (e.g., *α*1 and *α*5) and collagen IV (col IV)^1,4,11–17^. At this interface, oxygen diffuses from the lumen of the alveolar acini into the capillary plexus to be transported to the rest of the body. Disruptions in the homeostasis of the alveolocapillary barrier are considered hallmarks of diseases such as bronchopulmonary dysplasia (BPD), chronic obstructive pulmonary disease (COPD), and acute lung injury^8,10,18,19^. Whether of endogenous or exogenous origin (e.g. infection), respiratory diseases can cause severe clinical symptoms and are some of the leading causes of morbidity worldwide^20,21^. Therefore, understanding and re-inducing the mechanisms of alveologenesis would have a significant impact on regenerative medicine, developmental biology, and the management of pulmonary diseases. Beyond disease-related damage, the alveolar region is also a site that responds to airborne pathogens or other inhaled toxicants, as well as a drug administration route, further broadening the applications of representative alveolar models. A range of in vitro alveolar-capillary models have been used to study exposure to aerosols from electronic cigarettes^22^, nano-carriers (e.g., polyethyleneimine)^23^, TiO_2_ and ZnO nanoparticles^24^, inflammation^25^, or injury^26^.

Due to the limited availability of human lung tissue as well as ethical complications in its use, the majority of our current understanding of the respiratory system comes from animal models^9^. However, the high cost, ethical issues, and, in the end, limited translation to humans, has turned the focus towards engineering in vitro models of the lung tissue. Unfortunately, so far in vitro models of the alveolar unit have failed to closely recapitulate the structural complexity of alveolar tissue. Among the most common approaches are semi-2D cultures of alveolar cell monolayers^25,27–29^ that do not mimic the acinar 3D architecture of the native tissue, and organoids^30–32^ that although offering a 3D environment, lack cell-ECM interactions and are often reported^30,33,34^ to fail to maintain the ATI cell phenotype, and thus do not fully model adult alveolar tissue. Given that none of these approaches has, to our knowledge, achieved spontaneous formation of alveolar lumens lined by ATI and ATII cells, alternative routes are taken, in order to model the 3D morphology of the alveolar tissue in vitro. In these cases^25,35,36^, a dented/hemispherical substrate is used to shape the culture, enforcing a certain level of mimic of the accinar alveolar arrangement.

Given the advantages and limitations of the available systems, it is not hard to infer that choosing the appropriate cell source in combination with the right substrate for cell culture is critical for the success and range of applications of the engineered alveolocapillary model. Immortalized cell lines are often preferred^11,22,23,26,28,37–40^ due to high reproducibility and the practical limitations that come with using human primary cells (i.e., sparse healthy source material, complicated isolation protocols, limited passaging capacity, donor variability, and ethical concerns)^9,41^ and stem cell-based organoids (i.e., long differentiation protocols, dependency on Matrigel™, and lack of easy access to the apical epithelial side)^33,34^. On the other hand, immortalized cell lines usually fall short in recapitulating functions of the alveolar epithelium. They are therefore, so far, considered poor candidates for models that study epithelial function such as solute transport and metabolism, as well as injury and hrepair^9^, where primary sourced cells are preferred instead^25,36,42,43^. This study aims to bridge this gap by demonstrating that the performance of the established immortalized cell line NCI-H441 (H441) can be expanded to model alveologenesis-like events in addition to previously reported^37,44,45^ functions of native alveolar tissue, such as barrier function and surfactant protein secretion, when cultured on a physiologically relevant basement membrane mimic. H441 cells have characteristics of ATII cells and have been reported to outperform other immortalized cell lines^37,45^ while being able to form tight junctions, produce surfactants, polarize, and transdifferentiate into ATI cells^9,37,44,45^.

In most previous similar approaches, the alveolar cells were cultured on either side of a synthetic filter (e.g., TC insert) used as a divider separating epithelial and endothelial cells. Several materials of synthetic or biologic origin have been successfully prepared into membrane replicas. Some common synthetic materials include polycaprolactone (PCL)^11,23^, polydimethylsiloxane (PDMS)^46^, polycarbonate^35^, and PET^47^. Despite being considered cytocompatible, synthetic polymers are often inert to the cells and therefore often need surface coating with extracellular matrix (ECM) proteins to facilitate cell adherence and growth^18^. Further, most synthetically made membranes are plastic sheets with arrays of pores^46,47^, which come far from emulating the biochemical complexity and continuous porosity of the epithelial basement membrane. Membrane replicas have also been made from biopolymers, such as collagen and elastin composites^36^, and Matrigel™^24^. However, most casting processes of biological polymers into membranes result in relatively thick (> 4 µm)^36^ constructs not mimicking the in vivo epithelial-endothelial distance a fact that could complicate cross-talk between the juxtaposed cells. In addition, the use of undefined biomaterials (e.g., Matrigel™) has been associated with batch-to-batch variation and undefined composition.

FN-silk is a recombinant silk protein functionalized with an RGD-containing motif from fibronectin (FN), known to promote cell adhesion, proliferation, migration, and differentiation^48–50^. FN-silk can self-assemble into a membrane, yielding a fully defined, approximately 1 µm thin, fibrillar, basement membrane mimic, shown to be an excellent matrix for modeling barrier tissues, such as the blood vessel wall^51^, skin^52^, renal^53^, and blood–brain barrier^54^. Together, FN-silk membrane’s properties address key shortcomings of other systems regarding thickness, biomaterial origin, and substrate-cell interactions. Further, the FN-silk membrane is strong enough for handling, highly elastic^51,55^, and has been shown to outperform commercial PET membrane-based systems when it comes to modeling different barrier tissues^51–53^. Lastly, it is prepared simply by exploiting the unique property of the FN-silk protein to self-assemble into a tight mesh of fibrils at the air-liquid interface^55–57^.

In this study, immortalized epithelial (H441) and primary pulmonary endothelial (HPMEC) cell lines were cultured on the apical and basal sides of the FN-silk membrane, respectively, to develop an alveolar-capillary model. Our hypothesis was that the unique properties of the FN-silk membrane, which mimic key features of the basement membrane, could support H441 cells in performing essential alveolar functions—including barrier formation, surfactant production, and transdifferentiation into ATI-like cells—more effectively than conventional TC inserts. Notably, our system also enabled H441 cells to participate in alveologenesis-like processes, a previously unreported function of the cell line, while being the first, to our knowledge, build-up approach modeling aspects of alveologenesis in vitro.

## Results

### Study rationale

**Fig. 1:**
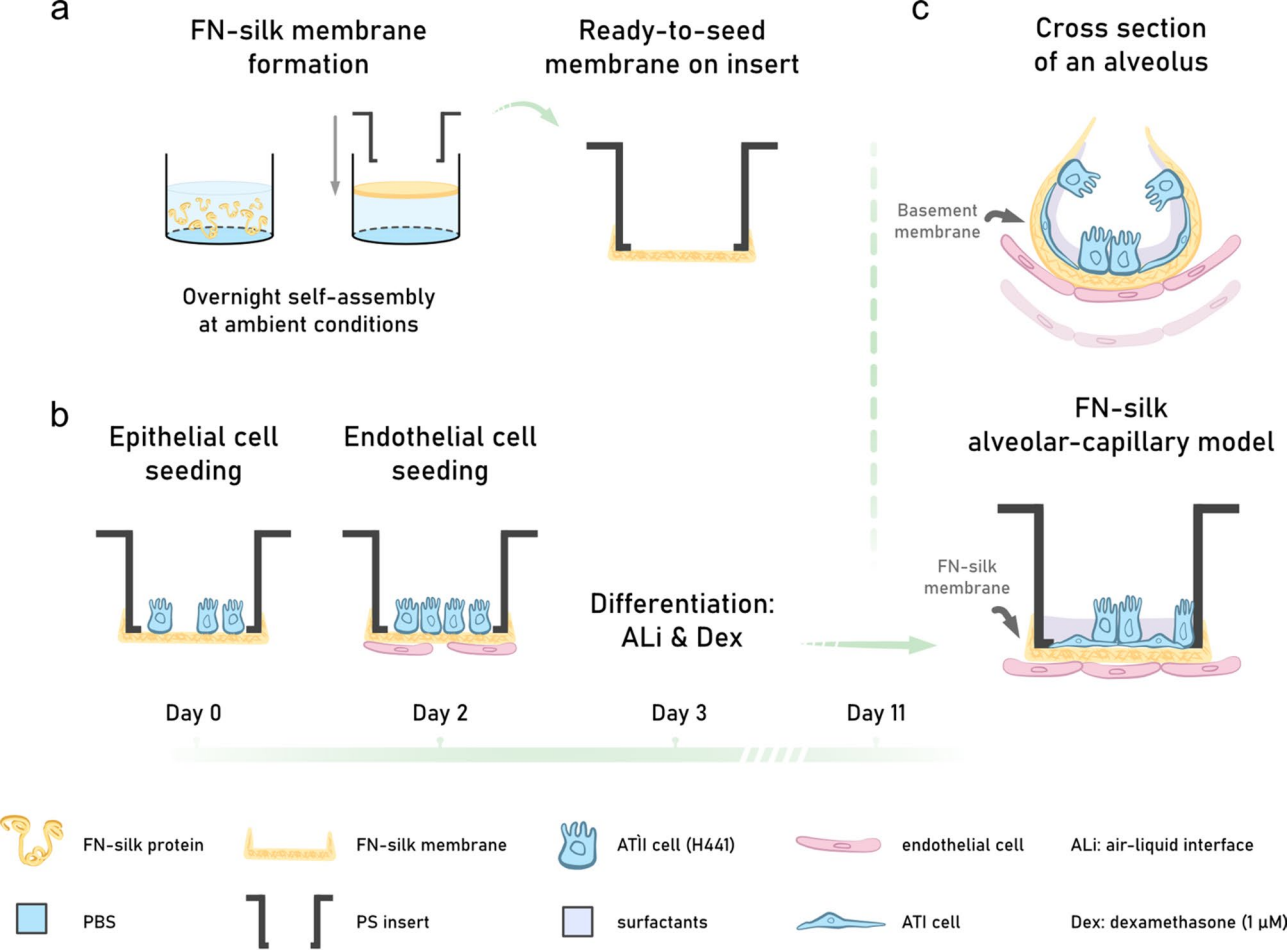
Schematic illustration of the procedure for making a FN-silk based alveolar-capillary model. (**a**) FN-silk solution self-assembles overnight forming the FN-silk membrane at the liquid-air interface. An empty insert is lowered onto the membrane, which attaches to the insert and can be lifted from the interface, yielding an FN-silk membrane-insert culture system. (**b**) Timeline showing important time points throughout the culture period, highlighting the seeding of the two cell types, and initiation of differentiation. (**c**) Illustration of an alveolus at the saccular stage and of the FN-silk alveolar-capillary model at the end of the culture period at Day 11.

Herein we investigated the potential of the FN-silk membrane as a substrate to support a physiologic-like alveolar-capillary model based on the H441 cell line, using the procedure illustrated in Fig. 1. Furthermore, we explored how the use of the FN-silk membrane can expand the potential of H441 cells, together with endothelial cells, for the construction of a model of the alveolar barrier. Our system was compared to commonly used commercial PET membrane-insert systems (i.e., TC inserts, Sarstedt).

### Cell viability and morphology

**Fig. 2:**
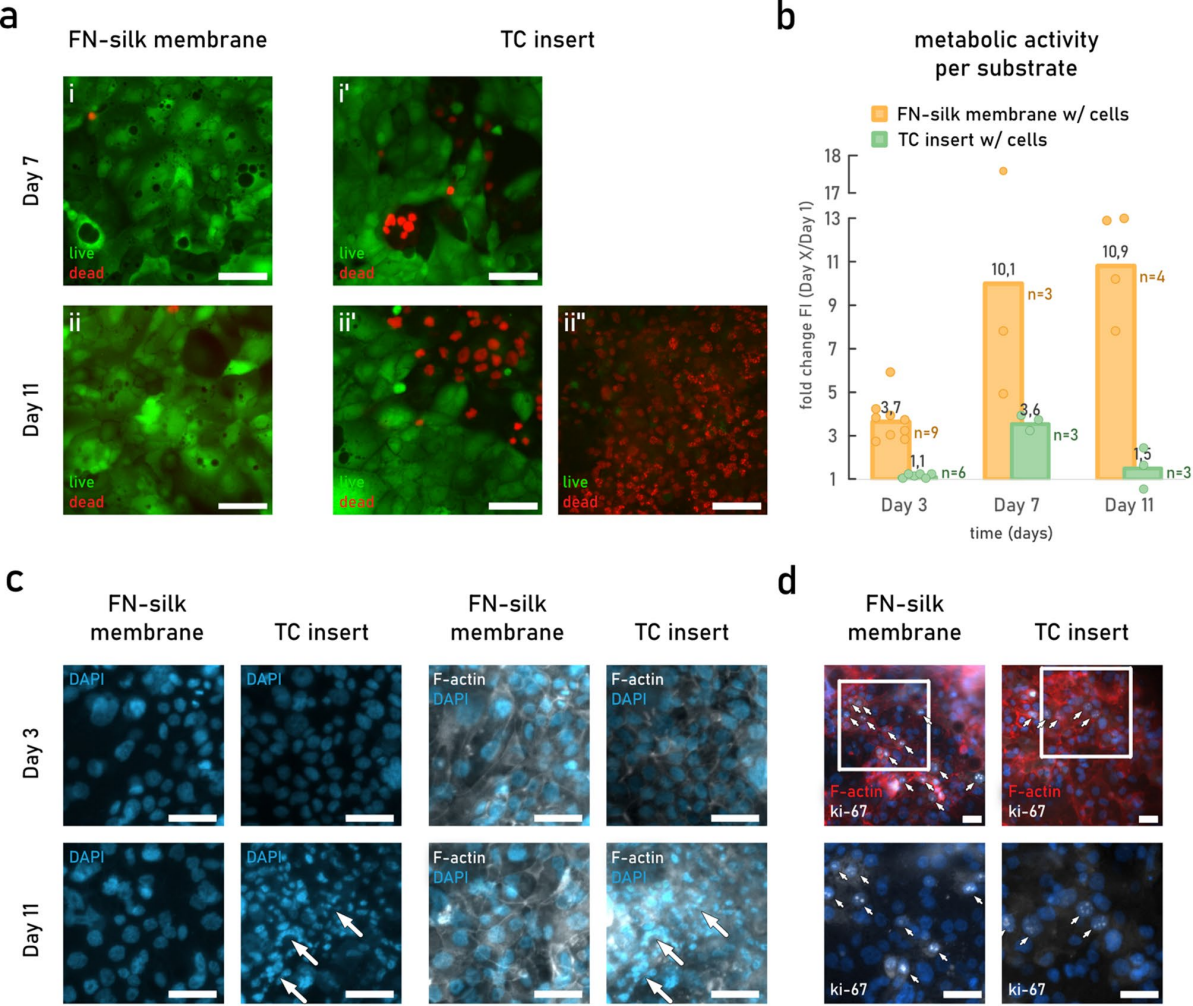
Epithelial cell viability and morphology. (**a**) Evaluation of epithelial cell viability on Days 7 (i, i′) and 11 (ii, ii′, ii″). when cultured on the FN-silk membrane (N = 1, n = 2) (i–ii) or the TC insert (N = 1, n = 3) (i′–ii′, ii″). Live cells are colored in green and dead cells in red. (**b**) Change of cell metabolic activity (AlamarBlue™) reported as a fold change (Day X/Day 1) of the measured fluorescence intensity (FI) throughout the culture period (Day X, X = 3, 7, 11) compared to Day 1. (n ≥ 3), (mean ± SD). FI was measured in a.u. and for each of the substrates the measured values were normalized to the respective culture area (a.u./cm^2^). (**c**) Cell morphology on Days 3 (top) and 11 (bottom) of epithelial cells on the FN-silk membrane or the TC insert. Left: Only the nuclei are stained (blue). Right: overlay with F-actin (white). Clusters of dead cells on the TC cultures are indicated (arrows). (**d**) Examples of proliferative epithelial cells on the FN-silk membrane or the TC insert at Day 11 (Ki-67, white) indicated with arrows. Top line: overlay with F-actin (red). Bottom line: zoomed in detail. Scale bars: 100 *µ*m (**a**), 50 *µ*m (**c**,**d**).

First, we evaluated the ability of the FN-silk and TC inserts to support viable cultures for 11 days. When cultured on the FN-silk membrane, the epithelial cells demonstrated excellent viability throughout the culture period (Fig. 2a.i–ii), and a metabolic activity consistent with an increasing-proliferating state until Day 7, followed by a plateau state until Day 11 (Suppl. Fig. 1). On TC inserts, on the other hand, parallel cultures demonstrated good viability until Day 7 (Fig. 2a.i′), but after this, viability declined considerably. Specifically, H441 viability was highly heterogeneous within the same TC insert, with certain areas maintaining some viable cells (Fig. 2a.ii′) while others contained clusters (Fig. 2c marked with arrows) or extended areas of dead cells (Fig. 2a.ii″).

A simultaneous decline in the metabolic activity values was observed (Suppl. Fig. 1), which featured a sharp decrease between Day 7 and 11 for cultures on TC insert. The different performance of the cells on the two substrates was more clearly reflected when visualizing the fold change of the metabolic activity compared to Day 1 (Fig. 2b). Already at Day 3, cells grown on FN-silk demonstrated a 3.7-fold increase in their metabolic activity as opposed to cells cultured on the TC inserts, where the increase was negligible (1.1-fold). On Day 7, the difference was more prominent, with the cultures on FN-silk showing a ten-times higher (10.1-fold) metabolic activity, which they maintained until Day 11 (10.9-fold). In contrast, cultures on TC-inserts reached a maximum of 3.6-fold increase of their metabolic activity at Day 7, which then decreased to the same range as in Day 1 (1.5-fold), further indicating the lack of a physiologic-like state of these cultures. Both cultures retained epithelial cells with the capacity to proliferate at the end of culture (Day 11), as indicated by the positive Ki-67 staining (Fig. 2d), an observation largely expected of an immortalized cell line.

The metabolic activity and viability trends were in agreement with the morphology of the cells and the overall performance of the cultures throughout the culture period (Suppl. Fig. 2). On the FN-silk membrane, the epithelial H441 cells appeared at lower numbers at Day 1 compared to the TC inserts, but reached confluence by Day 3 (Suppl. Fig. 2a.i–ii, b.i–ii). At this time point, the FN-silk based cultures began to show signs of stratification (Suppl. Fig. 2a.ii), with epithelial cells of both the typical cuboidal ATII-like and flattened ATI-like morphologies (Suppl. Fig. 3). In contrast, TC insert-based cultures exhibited a clearly different behavior that appeared to focus only on proliferating into a tightly packed monolayer (Suppl. Fig. 2b.ii). Over the course of the differentiation phase (Day 3–11), the two cultures continued to develop, each following its respective trend. Stratification on the FN-silk membrane continued and expanded over the entire culture area (Suppl. Fig. 2a.iii–iv), with cells maintaining an expanded morphology (Fig. 2c). In contrast, when cultured on the PET membrane, H441 cells failed to stratify, and had already on Day 7 started to shrink in size (Suppl. Fig. 2b.iii). By Day 11, they featured drastic decrease in size as well as undefined cell shape with loss of morphology, indicating an overcrowded culture and cell death (Fig. 2a.ii”, c, Suppl. Fig. 2b.iv) which was also reflected in the measured metabolic activity (Fig. 2b, Suppl. Fig. 1). To further challenge the FN-silk-based cultures, we evaluated cell viability after prolonged culture (Day 19). A fully viable culture with minimal cell death and cell morphology comparable to earlier time points was observed on Day 19, highlighting (Suppl. Fig. 4a, b) that the superior viability is owed to the favorable interaction of the epithelial cells with the FN-silk membrane and not due to a potential time-dependent artifact. In contrast, the clusters of dead cells on TC inserts, appeared to increase in size, covering almost the entire surface area of the PET membrane on Day 19 (Suppl. Fig. 4a, b), a finding consistent with the non-recoverable demise of these cultures that was detected already at earlier time points. Despite the evidence that maintaining an epithelial layer with minimal cell death on the TC insert-based cultures was not possible in the current setup, these cultures featured a viable epithelial layer (Fig. 2a.ii′) that contained proliferative cells (Fig. 2) and had a detectable metabolic activity (Fig. 2b, Suppl. Fig. 2) at Day 11. They were therefore deemed acceptable for further analysis regarding alveolar barrier and epithelial cell marker expression as described in the upcoming sections. Endothelial cell viability was also evaluated on single cultures (i.e., HPMEC monocultures) on the basal side of either substrate. Endothelial cells remained viable on both the FN-silk membrane and the TC insert (Suppl. Fig. 5) throughout the 9 days long culture period, which matched the time frame the endothelial cells are part of the co-culture (i.e., H441-HPMEC culture). In addition, endothelial cells were viable even after 19 days of culture on both substrates (Suppl. Fig. 4a,c), despite the widespread epithelial cell death seen on the TC inserts at this time point.

To investigate whether H441 cell viability is improved due to the biochemical properties of the FN-silk protein having a cell adhesion motif, or due to the effects of the FN-silk membrane as a complex, elastic, and fibrillar substrate, TC inserts were coated with FN-silk as previously described^48,58^ and seeded with H441 cells (Suppl. Fig. 6). During the early stages of culture (Days 1 and 3), some contribution from the FN-silk coating was observed in the morphology of the epithelial cells, with some cells showing flattened cell morphology and large nuclei (Suppl. Fig. 6), comparable to those cultured on the FN-silk membrane (Fig. 2c). However, by Day 7 this morphology was no longer observable, suggesting that the substrate was not suitable for supporting the development of the flattened ATI cell morphology. By Day 11, clusters of dead cells appeared, similar to the uncoated TC inserts (Suppl. Fig. 6), revealing the more complicated interaction of epithelial cells with the FN-silk membrane, possibly due to its mechanical and structural properties that will be described in the following section.

### Epithelial barrier formation

**Fig. 3:**
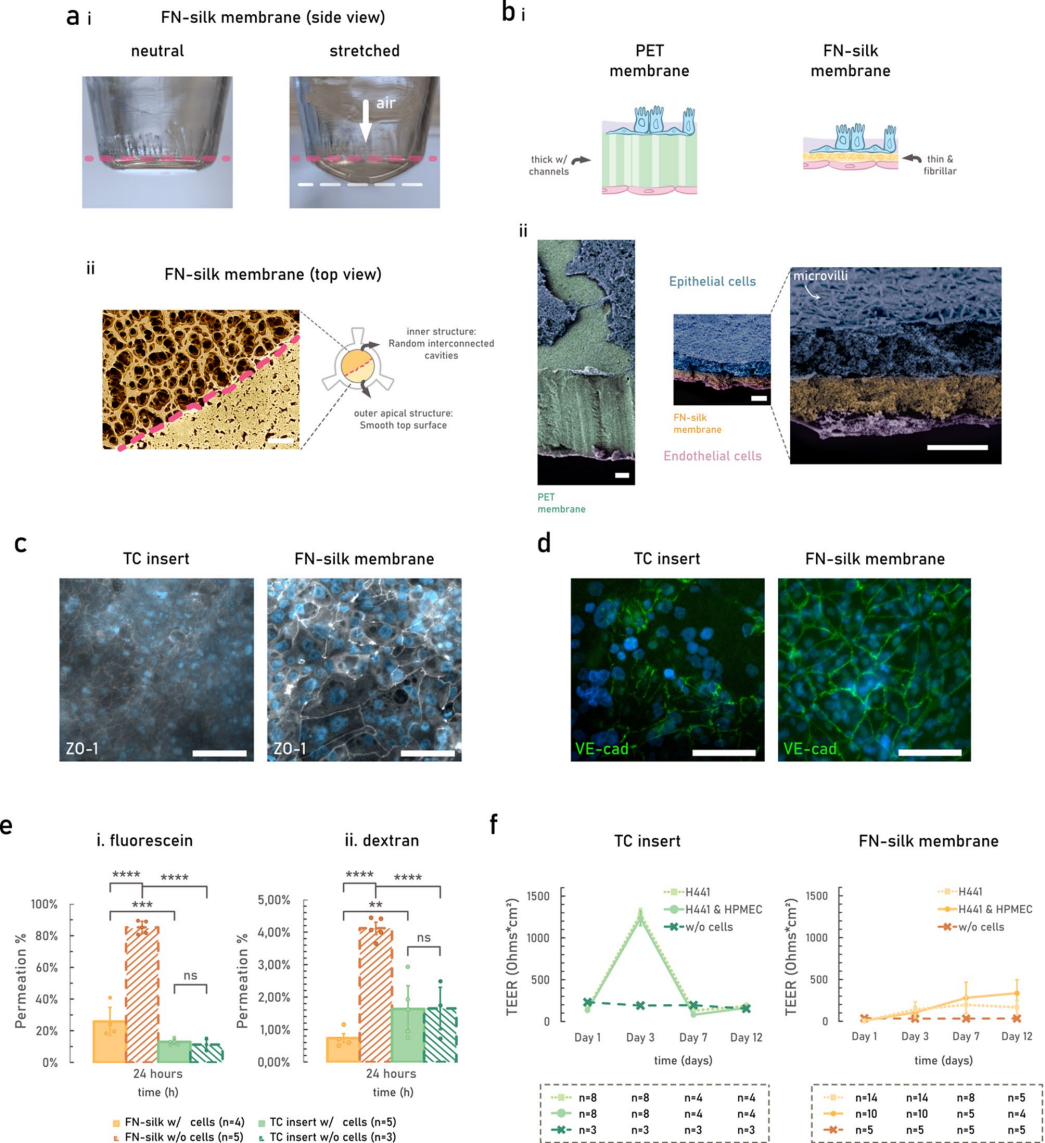
(**a.i**) Macroscopic images (side view) of the FN-silk membrane adhered to the bottom of an insert at a neutral state (left) and at a stretched-out position (right) after applying pressure through an air column. The magenta line indicates the starting position and the white line, the stretched-out position. (**ii**) False colored SEM image revealing the texture of the FN-silk membrane (top view). The dashed line (magenta) separates the areas before (bottom) and after (top) focused ion beam (FIB) etching. The upper, “air” side of the membrane has a smooth, less textured appearance, followed by an internal mesh structure of random interconnected cavities. (**b**) Scaled schematic (**i**) and false colored SEM (**ii**) images showing cross sections of the alveolar model based on a PET membrane (green) or the FN-silk membrane (yellow) on Day 11. The epithelial (blue) and endothelial (pink) layers are visible. (**c**) Immunofluorescence stainings for tight junctions (ZO-1, white) of the alveolar epithelial cells cultured on the TC insert (left) (*N* = 2, *n* = 1,2) or on the FN-silk membrane (right) (*N* = 4, *n* = 2,2,1,1) on Day 11. (**d**) Immunofluorescence stainings for adherence junctions (VE-cadherin, green) of the alveolar endothelial cells cultured on the TC insert (left) (*N* = 1, *n* = 1) or on the FN-silk membrane (right) (*N* = 1, *n* = 1) on Day 11. (**e**) Overnight (Day 11–12) permeation of fluorescein (376 Da) (**i**) and 70 kDa dextran (**ii**) through the FN-silk membrane (yellow) and TC insert (green) with (filled bars) or without (striped bars) cells (n ≥ 3), (mean ± SD). For the statistical analysis, a two-way ANOVA with Tukey’s multiple comparisons was performed. ns: non-significant, **p ≤ 0.01, ***p ≤ 0.001, ****p ≤ 0.0001. **f)** TEER measurements on alveolar models (i.e., epithelial-endothelial co-cultures and epithelial monocultures) cultured on FN-silk (right) or TC insert (left) for 12 days. The baseline of each substrate without cultured cells is also indicated (dashed line). Scale bars: 500 nm (**a.ii**), 2 µm (**b**), 100 µm (**c**,**d**).

Given the barrier properties of alveolar tissue, a functional epithelial barrier is crucial for a successful alveolar-capillary in vitro model. The FN-silk membrane (Fig. 3a) is composed of an elastic mesh of fibrils^51,55^ that facilitates proximity between the cell types cultured on either side (Fig. 3b.i–ii). Unlike stiff PET membranes found on TC inserts, the FN-silk membrane can stretch and bulge (Fig. 3a.i) in response to pressure difference (e.g., applied with an air column^51,55^. Importantly, the two substrates also differ in thickness and composition. The PET membrane is thicker (approximately 12 µm^53^) and solely permeable through etched pores (Fig. 3b.i–ii, Suppl. Fig. 7b), whereas the FN-silk membrane is significantly thinner (approximately 1 µm) and consists of a mesh of interconnected FN-silk protein fibrils with cavities in between (Fig. 3a.ii, Suppl. Fig. 7a).

The FN-silk-based alveolar-capillary models displayed excellent barrier properties confirmed with different methods (i.e., immunofluorescence, TEER, and passive permeation). First, the formation of continuous tight (ZO-1) (Fig. 3c, Suppl. Fig. 8) and adherence (VE-cadherin) (Fig. 3d) junction network was observed. Second, the functionality of the barrier was evaluated with passive permeation (Fig. 3e), as well as measurements of TEER (Fig. 3f). In contrast, ZO-1 expression was lower in cultures on TC inserts, where incomplete and disrupted epithelial (Fig. 3d) and endothelial barriers (Suppl. Figure 9a) were observed. The loss of barrier function on TC inserts was further verified by TEER measurements, which after an initial sharp rise on Day 3, decreased and stabilized in the same range as detected on Day 1, clearly demonstrating a failure of barrier integrity of the TC insert-based cultures. Similar sharp peak trends have also been observed in other models based on synthetic membranes^11,45^. Cultures on FN-silk on the other hand, showed a steady increase until Day 7, and then plateaued until the end of culture (Fig. 3f). ZO-1 stainings were performed at Days 3 and 7 (Suppl. Fig. 8a) to be evaluated together with the TEER measurements (Fig. 3f). They show a time-dependent formation of the tight junction network on FN-silk, starting from looser barrier (Day 3) and developing into a tighter on Day 7 (Suppl. Fig. 8a). The opposite was observed on TC-based cultures where the junctional network present at Day 3 had collapsed at Day 7 (Suppl. Fig. 8a). To explore whether endothelial cells might contribute to epithelial barrier formation in a manner detectable by TEER, single seeded membranes (i.e., epithelial cell monocultures) were also evaluated. A slightly lower TEER value was observed for the single-seeded epithelial FN-silk constructs, while no difference was detected on the TC inserts. However, ZO-1 expression in single seeded cultures showed no observable difference compared to the double seeded cultures (Suppl. Fig. 8b). The endothelial barrier formed equally well on single- (i.e., HPMEC only) and double-seeded FN-silk cultures, as concluded from the formation of tight and adherence junction networks, in contrast to the TC insert-based endothelial cultures that failed to form a complete endothelial barrier (Suppl. Fig. 9b).

To further evaluate the barrier function, passive permeation across of the alveolar-capillary models was investigated using fluorescein (376 Da) and dextran (70 kDa) as small and large molecule examples, respectively (Fig. 3e, Suppl. Fig. 10). The bare FN-silk membrane (i.e., without cells) showed higher permeability to fluorescein and a faster permeation rate compared to the bare PET membrane. Within 1 h, 20% of the loaded sample (66-fold more) passed through the FN-silk membrane, while the PET membrane remained more or less impermeable (0.3%) (Suppl. Fig. 10). After 4 h, more than half (59%) of the molecules had permeated through the FN-silk membrane, compared to 3% for the PET membrane (Suppl. Fig. 10). Following 24 h of incubation, 85% of the molecules permeated through the FN-silk membrane, i.e. 7-fold more than through the PET membrane (11%) (Fig. 3e.i). The same permeation trend was observed when dextran was used instead, with the overall permeation being lower, given the molecule’s large size (Suppl. Fig. 10). After 24 h, dextran had permeated the FN-silk membrane at a 2.5-fold higher value (4.1%) than the PET membrane (1.7%).

Although bare FN-silk membranes were significantly more permeable to both molecules than PET membranes, a behavior that agrees with the substrates’ differences in thickness and composition (Suppl. Fig. 7), the presence of cells had a significant effect on permeation rate only for cultures on FN-silk. The FN-silk alveolar-capillary model significantly slowed fluorescein permeation by 3.3-fold (26%), and almost inhibited the permeation of dextran (0.7%) overnight, compared to the bare FN-silk membrane (Fig. 3e, Suppl. Fig. 10). This is in agreement with paracellular permeation being limited to molecules < 40 kDa for in vitro alveolar models as previously reported^9^. In the case of the TC inserts, no significant difference was detected between the membranes with and without cells, both for fluorescein (13% and 11% respectively) and for dextran (1.6% and 1.7%), demonstrating that the substrate, rather than the cell barrier, is the limiting factor for permeation in this case.

### Characterization of the alveolar epithelial layer

**Fig. 4:**
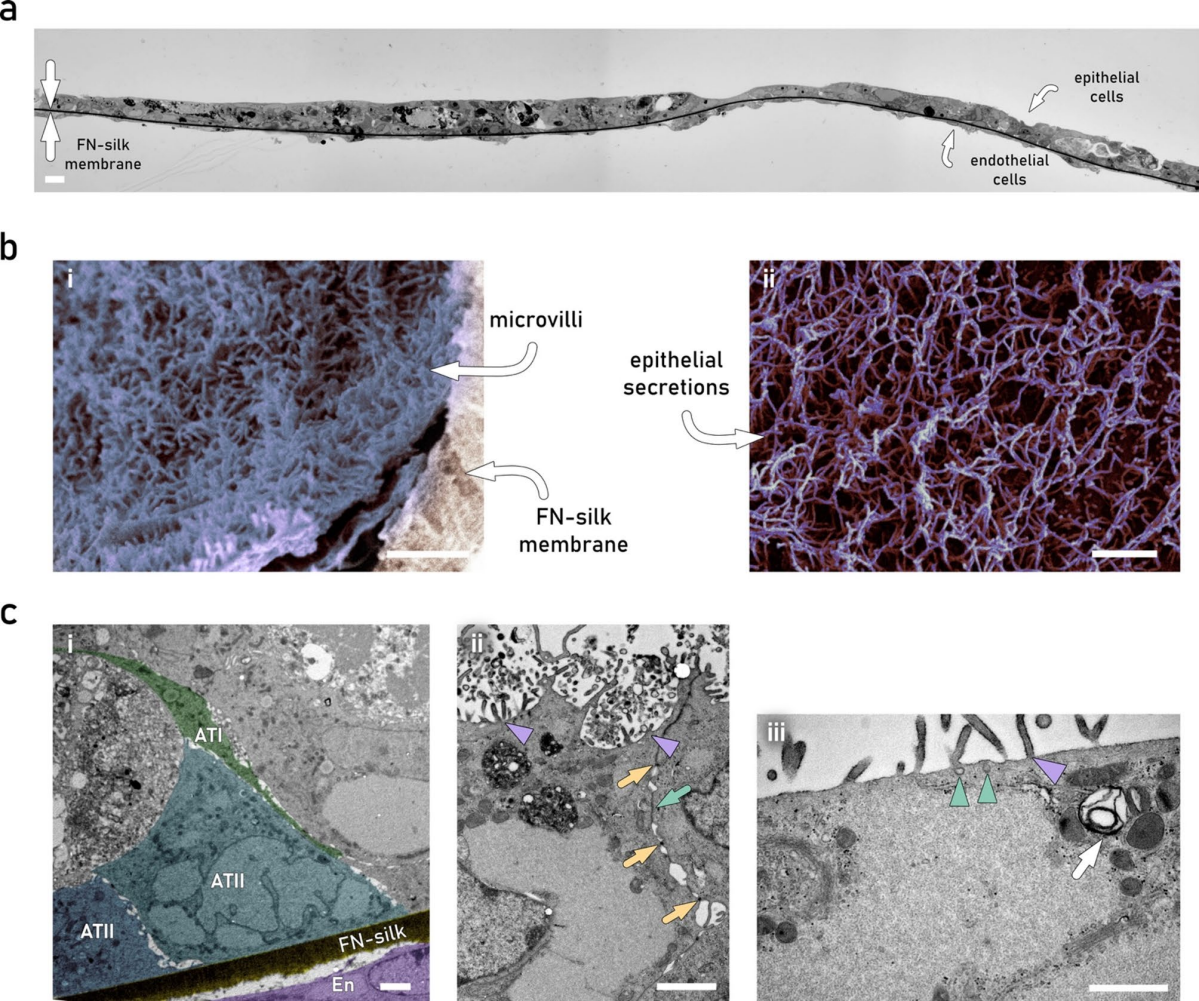
Characterization of epithelial H441 cells on the FN-silk alveolar-capillary model with TEM (**a**,**c**) and SEM (**b**) imaging on Day 11. (**a**) Cross-sectional overview, the epithelial and endothelial layers are visible on either side of the FN-silk membrane, seen here as thin black line. (**b.i**) Microvilli-rich apical side of the epithelial layer (tilted view, microvilli false colored blue, FN-silk membrane yellow). (**ii**) Extracellular epithelial secretions on the apical side (top view, false colored purple) (*N* = 1, *n* = 3). (**c.i**) Cross section showing false colored ATII-like (blue), ATI-like (green), and endothelial cells (purple) on the apical and basal sides of the FN-silk membrane (yellow), respectively. Ultrastructure of epithelial cells featuring: tight junctions (green arrow), desmosomes (yellow arrows), and microvilli (purple arrowheads) visible on the epithelial layer (**ii**) and caveolae (green arrowheads), lamellar body (white arrow), and microvilli (purple arrowhead) (**iii**) (*N* = 1, *n* = 2). Scale bars: 20 µm (**a**), 2 µm (**b**,**c.i,ii**), 1 *µ*m (**c.iii**).

In addition to its barrier properties, the alveolocapillary unit serves other key functions in vivo, such as surfactant production, ionic transport, and gas exchange. The capacity of the FN-silk-based alveolar-capillary model (Fig. 4a) to recapitulate these properties was evaluated using different imaging techniques (i.e., transmission (TEM) and scanning electron microscopy (SEM), and immunofluorescence imaged with widefield or confocal microscopy) to visualize morphology, ultrastructure, and protein expression. Due to the clear underperformance of TC insert-based cultures, in terms of cell viability, morphology, and barrier formation, it was deemed relevant to only evaluate the FN-silk-based cultures with some of the advanced imaging techniques hereafter.

**Fig. 5:**
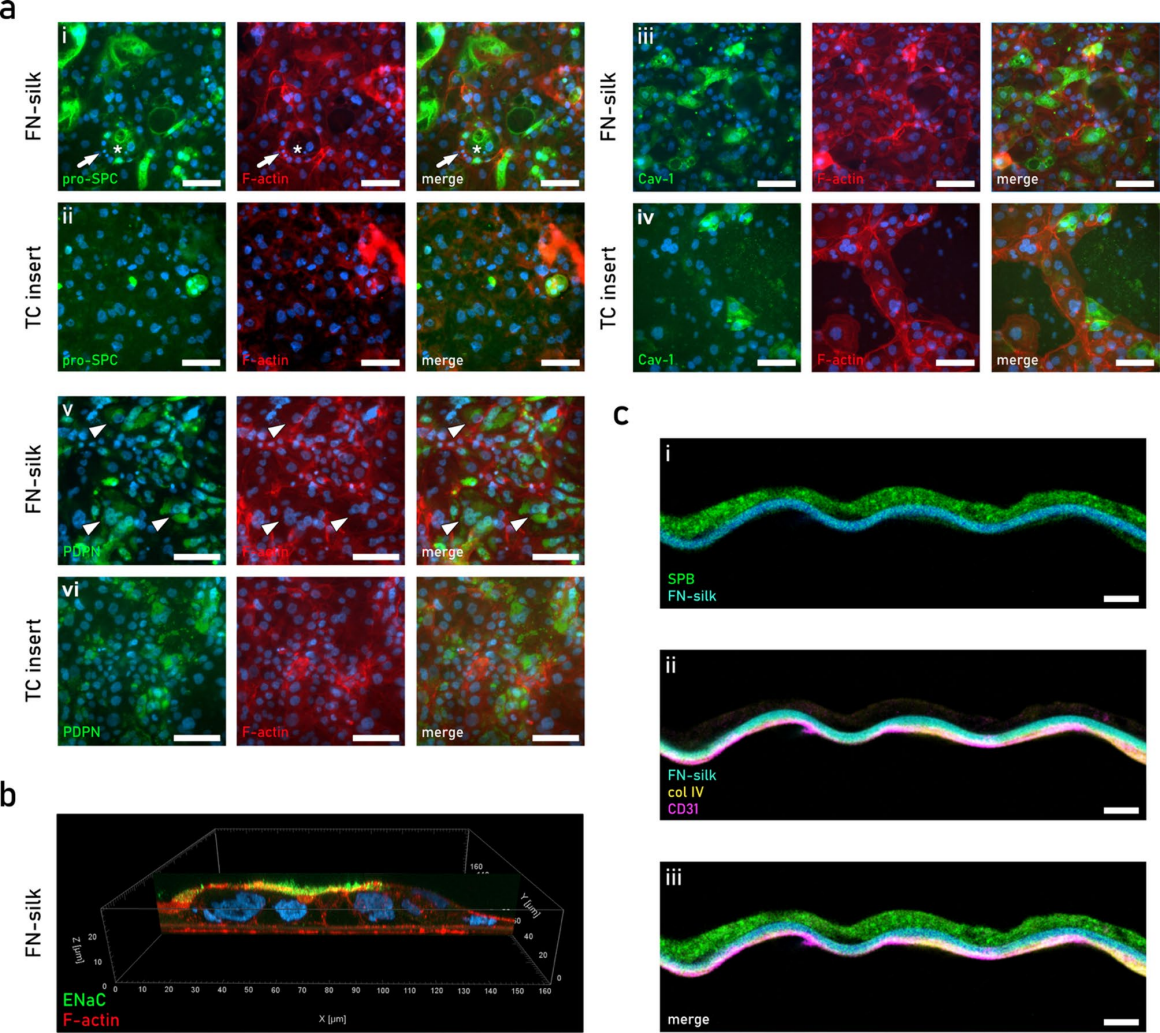
Typical alveolar epithelial marker expression and localization on the alveolar-capillary models based on FN-silk or TC inserts. (**a**) Immunofluorescence stainings of pro-SPC (green) (i–ii), caveolin-1 (green) (iii–iv), and podoplanin (green) (v–vi) of alveolar epithelial cells on Day 11 cultured on the FN-silk membrane (i: *N* = 3, *n* = 2,1,1, iii: *N* = 3, *n* = 2,1,1, v: *N* = 1, *n* = 1) or the TC insert (ii: *N* = 3, *n* = 2,1,1, iv: *N* = 2, *n* = 1, vi: *N* = 1, *n* = 1). Cuboidal ATII-like cells (arrow) surrounding a pro-SPC-rich area (asterisk) (**a**) are marked. PDPN-positive cells with ATI-like morphology (arrow heads) (**c**) are also indicated. (**b**) Immunofluorescence staining of ENaC (green) expressed on the apical side of the epithelial cells of the FN-silk-based model on Day 11 as detected in an orthogonal view (XZ-reslice of confocal image stack). Actin filaments are also stained (phalloidin, red). (**c**) Sagittal section of FN-silk-based models (Day 11) stained with multiplex immunofluorescence. SPB (green), col IV (yellow), and CD31 (magenta). Fluorescently labeled FN-silk was used to visualize the FN-silk membrane (teal). Nuclei are counterstained with DAPI (blue) (**a**,**b**). Scale bars: 100 *µ*m (**a**), 10 *µ*m (**c**).

H441 cells cultured on the FN-silk membrane exhibited the typical ATII and ATI morphologies (Fig. 4c.i) at the end of culture (Day 11), as also observed in earlier time points (Suppl. Fig. 3). They also featured structural, and functional characteristics of ATII and ATI cells, indicating that the FN-silk membrane can support both alveolar epithelial cell types. ATII cells were observed as tall, cuboidal cells (Fig. 4c.i) with a polarization distinctive by the presence of microvilli (Fig. 4b.i, c.ii, iii, Suppl. Fig. 11a.i–ii) as previously shown with the same technique in primary animal-derived cells^59^. Additionally, cell secretions were observed on the epithelial cell layer (Fig. 4b.ii, Suppl. Fig. 11a.iii), demonstrating that ATII cells on FN-silk membrane perform their secretory function. This is also corroborated by the presence of lamellar bodies (Fig. 4c.iii), i.e., intracellular compartments that are used for surfactant storage^9,60^. Further, pro-SPC, the most typical ATII cell marker^9^ was found in abundance on the FN-silk-based model using immunofluorescence (Fig. 5a.i) compared to its sparse expression on TC insert-based models (Fig. 5a.ii). This finding, together with the high expression of SPB also detected with immunofluorescence (Fig. 5c.i, iii) and the high amount of secretions observed with SEM (Fig. 4b.ii, Suppl. Fig. 11a), are indicative of H441 being in a more physiological-like state on the FN-silk membrane model where they can assume native-like ATII functions. Interestingly, the native-like morphology and spatial distribution of ATII cells^9^ can also be observed with immunofluorescence, where groups of cuboidal cells can be found next to pro-SPC-rich areas (Fig. 5a.i).

ATI cells can also be detected with immunofluorescence using caveolin-1 (Cav-1) and podoplanin (PDPN) markers. Caveolins are omega-shaped invaginations found on the apical and basal sides of the cell membrane in ATI cells^61^. In vitro, the loss of SPC expression and the upregulation of Cav-1 expression is considered typical of ATII to ATI transdifferentiation^9,61^. Cav-1 expression was found to be noticeably higher on H441 cells cultured on the FN-silk membrane (Fig. 5a.iii) as opposed to the TC insert (Fig. 5a.iv). Moreover, PDPN expression is a hallmark of ATI transition^62,63^, and although its expression was observable in cells cultured on both substrates (Fig. 5a.v–vi), on the FN-silk cultures (Fig. 5a.v) it often co-localized with large, flattened cells consistent with differentiated ATI cells. The same morphology was also observable with TEM (Fig. 4c.i), which in addition captured the presence of caveolae (Fig. 4c.iii) as previously seen in ex vivo samples^9,61^.

In vivo, epithelial sodium channels (ENaC) serve the crucial function of alveolar fluid clearance and are an indicator of epithelial cell polarization. Similarly, on FN-silk-based cultures, ENaC expression was observed on the apical side of the epithelial cells (Fig. 5b) using confocal microscopy. Other important ultrastructural components of the alveolar epithelium were detected on the FN-silk models, such as tight junctions (Fig. 4c.ii) also observed with immunofluorescence (Fig. 3c), and desmosomes (Fig. 4c.ii). Given that this work aims to investigate the importance of the cell-BM interaction for the engineering of more physiologically relevant alveolar models, we also evaluated the capacity of H441 cells to produce key BM markers. Such markers include col IV and laminins, both known to be important components of the alveolar microenvironment^12–14^, playing a critical role in coordinating alveolar morphogenesis^15,16^. As observed by immunofluorescence stainings, H441 cells expressed col IV on both substrates, with the expression on FN-silk being more abundant (Suppl. Fig. 12). On FN-silk membranes, basal expression of col IV by endothelial cells was further investigated and observed in cross sections of the FN-silk-based cultures (Fig. 5c.ii, iii). Interestingly, laminin (*α*1, *α*5) expression was observed only on the FN-silk-based models (Suppl. Fig. 13).

Although this work focuses on characterizing the double-seeded alveolar-capillary model, evaluating pro-SPC and Cav-1 expression on single-seeded cultures containing only epithelial cells gave us a better understanding of the contribution of the endothelial cells to the system. Both markers were present, but harder to detect in single seeded cultures (Suppl. Fig. 14), potentially indicating the importance of the endothelial-epithelial crosstalk for these functions.

### Alveologenesis and 3D alveolar structures

So far, we have demonstrated that the FN-silk-supported alveolar-capillary model can successfully mimic morphological, subcellular, and functional characteristics of the alveolar barrier tissue. In this section, we will describe that within this model, H441 cells can spontaneously self-organize into structures mimicking the 3D architecture of native alveolar tissue, by assuming functions that drive the process of new alveolar tissue formation—alveologenesis.

**Fig. 6:**
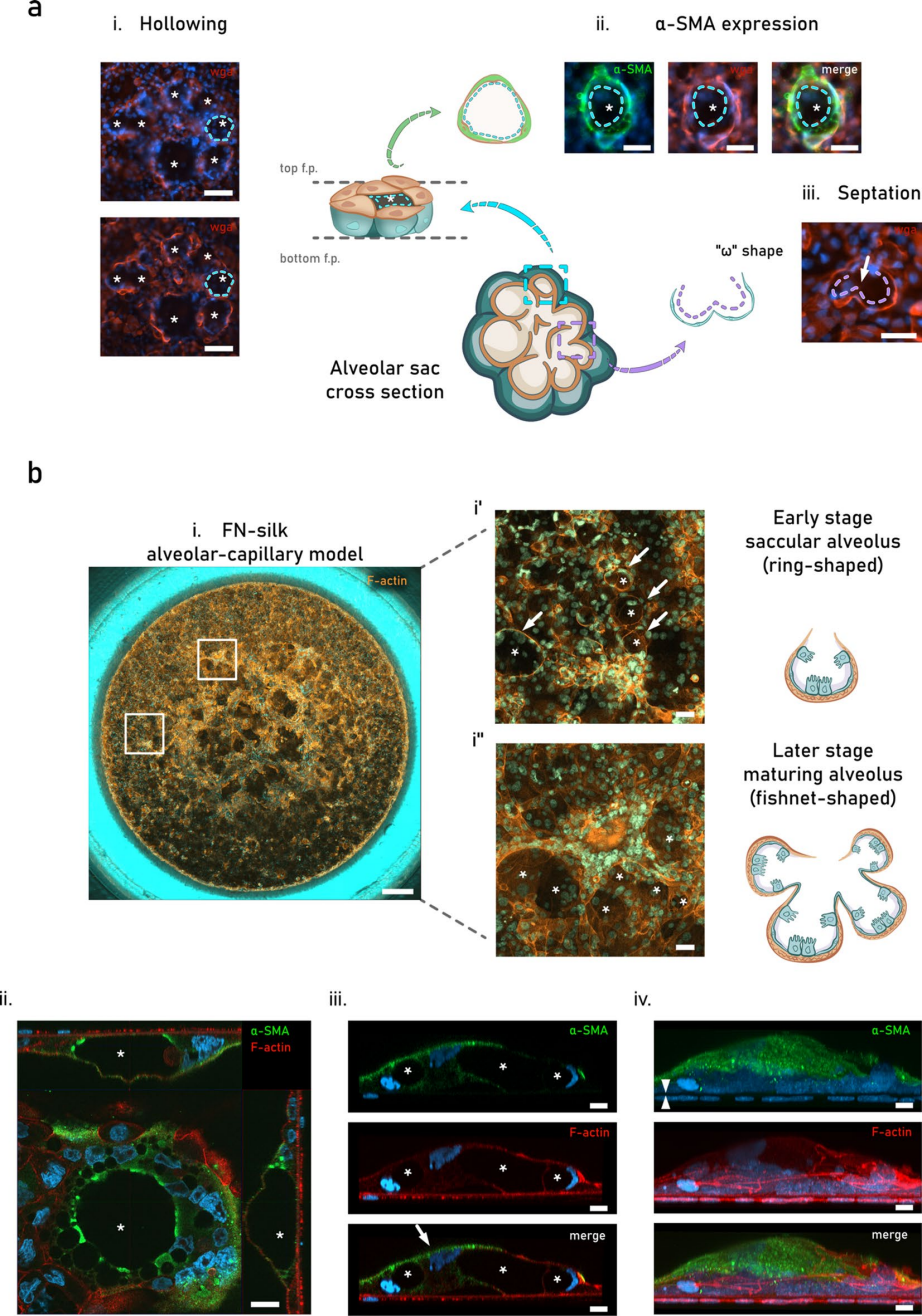
Epithelial cells assume native tissue-like 3D organization on the FN-silk alveolar-capillary model. (**a**) Three key concepts observed during alveologenesis (schematically illustrated) are also detected on the FN-silk model: (**i**) cell hollowing, (**ii**) *α*-SMA expression (green) (N = 3, n = 1), and (**iii**) septation. The secondary crest (tip) is indicated (arrow). Cells are stained with the glycoprotein wheat germ agglutinin (wga) (N = 1, n = 4) (red) (i–iii). (**b**) 3D alveolar formations observed on the FN-silk model stained with F-actin (N = 4, n = 2) on Day 11. (**i**) Overview of the entire construct and zoomed in details showing early stage, saccular (i′) or later stage, fishnet-shaped (i″) 3D alveolar conformations. Typical ring-shaped, saccular formations are indicated (arrows). Confocal Z-stacks of completely enclosed saccular-alveoli characterized by hollowing and formation of a lumen (asterisk) that is enclosed by epithelial cells: (**ii**) Orthogonal view (XY, XZ, and YZ-reslice), (**iii**) orthogonal view (XZ-reslice), and (**iv**) maximum intensity projection. An elongated cell contributes to 2 air-sac lumens (arrow) (ii) and *α*-SMA is expressed apically on the newly formed air-sac. The FN-silk membrane position can be seen as a thin non-fluorescent line (arrow heads) with the epithelial cells located apically and the endothelial cells visible basally. F-actin (orange (i), red (ii–iv)) and *α*-SMA (green (ii–iv)). Nuclei (blue) are stained with DAPI, Lumens are marked (*). Scale bars: 100 µm (**a.i**), 50 µm (**a.ii–iii**,**b**.i′–i″), 20 µm (**b.ii**), 10 µm (**b.iii–iv**)..

Live imaging of ex vivo lung tissue^64,65^ has revealed key cellular behaviors crucial for the formation of new alveolar tissue namely cell hollowing, septation, and *α*-SMA expression. When monitoring the 3D architecture, all these phenomena were observed in the alveolar-capillary model based on FN-silk (Fig. 6a). In contrast to their TC counterparts within this work and previously developed models^11,22,23,28,37,38,40^, H441 cells did not form a flat monolayer when cultured on the FN-silk membrane (Suppl. Fig. 2, Suppl. Fig. 3). Instead, they stratified (Suppl. Fig. 2), forming lumen-like 3D structures (Fig. 6) that mimic the native alveolar tissue architecture^64,65^.

The beginning of lumen formation was identified at early time points and continued to evolve as the alveolar-capillary model differentiation progressed. Signs of epithelial stratification were observable at Day 3 (Suppl. Fig. 2a.ii) and had clearly increased by Day 7 (Suppl. Fig. 2a.iii). After this first week, simple lumen-shaped structures could be identified as ring-shaped, 1–2 cell layer-tall formations composed of usually 2 or 3 connected cells, surrounding an emerging airspace (Suppl. Fig. 15). In fully differentiated cultures (i.e., Day 11), the lumen formations were more abundant and easier to locate (Fig. 6b.i,i′). The topology was consistent with the saccular stage of alveolarization^2^, an argument further supported by the presence of critical alveolarization processes (i.e., cell hollowing, a-SMA expression, and septation) (Fig. 6a), which are briefly described below. Ex vivo studies of lung explants reveal that the hemispherical arrangement of these airspaces forms as a result of cell hollowing, where a cavity first appears in a cell-dense area and then is lined by a layer of stratifying cells observable at a higher focal plane than the original one^64^. In order to facilitate tissue plasticity on these newly forming alveolar ridges, myofibroblasts locally produce *α*-SMA during a limited time window^2,65^. Herein, such structures were easy to identify with widefield microscopy by locating bright ring-shaped cell conformations at the top focal plane, combined with a hemispherical lumen space lined with cells at the bottom focal plane (Fig. 6a.i, b, Suppl. Fig. 15a). Notably, a clear and localized expression of *α*-SMA was observed on the ridges of the hollowed airspaces (Fig. 6a.ii, b.ii–iv), suggesting that, when cultured on the FN-silk membrane, the H441 cells can assume this function. We speculate that this localized *α*-SMA expression happens in connection to the other alveologenesis-like events described in this section, but the potential of e.g., unrelated epithelial to mesenchymal transition cannot be excluded. The formation of multiple cell-made luminal airspaces was also verified by the use of confocal microscopy which revealed that *α*-SMA-positive cells come together to form multiple lumens (Fig. 6b.iii). Interestingly, in some cases these lumens were entirely surrounded by cells, yielding enclosed alveolar air spaces with a hollow center (Fig. 6b.ii–iv, Suppl. video 1 and 2). Similar 3D formations were also observed in cross sections with TEM imaging (Suppl. Fig. 16).

Further down the process of alveologenesis, saccular alveoli give rise to new ones through the process of septation. That is, the formation of *ω*-shaped conformations, septa, formed by migrating cells that subdivide the original airspace into two new hollow ones. This process of secondary septation is described as a key step that eventually gives rise to the mature alveolar stage^2,64^. In the case of the FN-silk model, epithelial cells were observed to form septations (Fig. 6a.iii), a sign that the model could support even more complicated 3D alveolar structures. As the simple ring-shaped structures merged, more complicated formations appeared (Fig. 6a.i,b.i, i″) that consisted of multiple interconnected lumen-shaped airspaces coming together to form the typical fishnet pattern of the maturing alveolar tissue^2,65^.

On Day 11, the culture was dominated by the later stage, fishnet-shaped alveolar structures, but saccular conformations were still present (Fig. 6b), indicating that alveologenesis-related events take place continuously in the model. To further understand the contribution of the endothelial cells to the process, we omitted them and investigated the performance of the epithelial cells in single seeded cultures (Suppl. Fig. 15, Suppl. Fig. 17). In this case, simpler and more sparse saccular conformations were observed on Day 7 (Suppl. Fig. 15b), which increased in number by the end of the culture period (Suppl. Fig. 17). Saccular and more mature alveolar structures as well as septating cells (Suppl. Fig. 17) were present in the single seeded cultures on Day 11, although they were noticeably fewer and simpler compared to the epithelial-endothelial co-cultures. This suggests that the FN-silk membrane itself is a suitable matrix to facilitate alveologenesis-mimicking processes by H441 cells, but the addition of the endothelial cells is important in order to achieve more complete maturation within the same time frame.

## Discussion

We herein demonstrate that the FN-silk membrane is a more physiologically relevant BM-mimic than TC inserts for alveolar tissue modeling. It allows for the engineering of an in vitro alveolar-capillary model within which cells resume morphology and functions resembling those of the in vivo tissue, namely barrier formation, surfactant and ECM production, and processes reminiscent of alveologenesis.

The FN-silk-based alveolar-capillary model outperformed the TC insert model in barrier development, delaying the permeation of small molecules and nearly blocking larger molecules, in agreement with the trends of paracellular permeation in alveolar tissue^9^. The epithelial and endothelial barriers were evaluated using several methods, including microscopy, TEER, and passive permeation, all of which revealed a clear advantage for the FN-silk system. In addition to barrier properties, epithelial cells cultured on FN-silk membranes displayed typical ATI and ATII morphologies and expressed both type II (pro-SPC, SPB) and type I (Cav-1, PDPN) specific markers, either more (pro-SPC, Cav-1) or equally well (PDPN) as their TC insert counterparts. These findings indicate that a subset of the epithelial cells cultured on the FN-silk membrane differentiated into ATI-like cells, while others retained their ATII phenotype, similar to the native alveolar tissue epithelial cells. The presence of cell-secreted surfactants yields a more sophisticated alveolar barrier model, making it a relevant option for alveolar barrier studies (e.g., infection, intracellular or paracellular transport, inhalation pharmaceutics, nanoparticle or other air pollutant exposure, etc.). Further, the well-established endothelial barrier can enable studies on drug delivery through the vasculature, as well as on alveolar pathologies where endothelial barrier disruption is critical (e.g., acute lung injury or pulmonary edema)^19^.

We attribute the advantages of the FN-silk-based alveolar capillary models to key distinctions from commercial tissue culture systems with PET membranes (e.g., TC inserts). To begin with, FN-silk is composed of a silk protein functionalized with an RGD motif rather than an inert synthetic material. It has been found to positively affect key cellular functions (i.e., adhesion, migration, proliferation, and differentiation)^48–50,52,53,66^ for a range of different cell types. However, as revealed by coating the TC insert membrane with FN-silk, it requires more than the presence of the FN-silk protein in the cell microenvironment to have a significant impact on cells. This observation strengthens the notion that cells depend on complicated biomechanical cues from their ECM to assume in vivo-like functions, something that coating solutions often fail to provide. The FN-silk membrane, on the other hand, is a micrometer-thin fibrillar matrix with a complex internal architecture of interconnected nanometer-wide fibrils interspersed with cavities. As a result, unlike the approximately 10 times thicker PET membrane, the FN-silk membrane does not rely on uniform, straight pores for communication between the apical and basal sides, but facilitates better crosstalk across bilaterally seeded cell layers^51^. In addition, as demonstrated here, and further investigated in previous works^51,55^, the FN-silk membrane has exceptional elasticity, a property highly relevant in the case of the alveoli^67^, given the dynamic nature of the tissue during breathing. Although the individual contribution of each of these FN-silk membrane properties cannot be discerned, we believe that their combination contributes to a more physiologic-like cell-substrate interaction, allowing epithelial cells to assume complicated functions such as the herein demonstrated ECM production and stratification, rather than uncontrolled proliferation. We therefore propose that this in vivo-like cell activity facilitated H441 epithelial cells to also assume alveologenesis-related functions on the FN-silk membrane. Further, the epithelial cells showed morphological differences corresponding to ATI and ATII cells early on when cultured on the FN-silk membrane, reflecting a more in vivo-like phenotype than in the TC insert-based cultures. TC inserts allowed for H441 adhesion and proliferation, but there was no evidence of a stratifying cell arrangement. These differences were also reflected in the metabolic and TEER profiles of the two substrates, with cultures on FN-silk going through a gradual growth phase that took place in parallel with stratification and formation of, at first, ring-shaped saccular conformations followed by fishnet-shaped maturing ones overtime. In addition, the co-cultured cells produced both key BM components (i.e., col IV and laminins)^12–14^. Epithelial cells produced laminins (*α*1 and *α*5), and both endothelial and epithelial cells produced col IV. Laminin production was detected exclusively in FN-silk cultures, which we hypothesize may be attributed to the critical role of integrins in laminin polymerization^14^, given that FN-silk allows for integrin-mediated cell adhesion^48^.

The alveolar-capillary model we developed is a straightforward system consisting only of the FN-silk BM-mimic, the immortalized cell line H441, widely accepted as a well-performing ATII cell line for in vitro culture^9,37,44,45^, and a primary endothelial cell monolayer. Despite its simplicity, our system enables processes considered key for alveologenesis, namely cell hollowing and alveolar lumen formation, septation, and *α*-SMA expression localized on the alveolar ridges; a surprising finding, given that in vivo this function is carried out by myofibroblasts. This is the first time, to our knowledge, such processes have been demonstrated to this extent in vitro, as they so far have been studied and understood mostly using animal models^15,64,65,68,69^. This achievement opens up promising avenues for alveolar tissue engineering as it allows for the modeling of the alveolar 3D architecture in vitro using human cells. This aspect has been so far either completely omitted in similar systems^25,27–29^, or enforced using concave culture substrates^25,35,36^. When it comes to stem cell-based organoid approaches, promising advancements have been made^30–32^, reporting spontaneous alveolar morphogenetic processes such as budding^30,31^, and branching^30^. However, these models still lack the capacity to mimic the events of the alveolarization stage of lung development characterized by both the maturation of epithelial cells into the ATI and ATII phenotypes, as well as the formation of alveolar lumens during alveologenesis, appearing to face a trade-off between epithelial cell maturation and achieving a more complicated 3D architecture. Given that no other approach of mimicking the alveolar-capillary barrier has, to our knowledge, so far demonstrated immortalized epithelial cells assuming cellular arrangements other than monolayers^9,37,44,45^, we herein demonstrate that H441cell line limits can be expanded when cells are cultured on physiologically relevant matrices, like the FN-silk membrane, making them better tools and improving their impact on early in vitro research.

Despite demonstrating that H441 cells can participate in alveologenesis-like processes, nullifying the limitations of an immortalized cell line is an unrealistic expectation. Despite their common use to model the alveolar epithelium, H441 cells, given their immortalized origin, inherently lack the capacity to fully recapitulate all in vivo functions, while their adenocarcinoma origin could drift them away from phenotypes that faithfully represent alveolar physiology, affecting, for example, their tendency to uncontrollably proliferate and eventually die when cultured on the TC inserts. With our system’s restrictions in mind, completely isolating the contribution of the FN-silk membrane from the potential phenotypical plasticity of H441 cells is impossible without also evaluating how primary alveolar cells would perform when cultured on the same substrate. Considering this limitation, a conclusive interpretation of the *α*-SMA expression in our system is not possible within the scope of this work. Although the timing (i.e., on early-stage saccular-like conformations) and localization (i.e., apical side of forming ridges) of its expression in our model correlate with those reported on in/ex vivo alveologenesis^2,65^, further studies are required to investigate a potentially causal relationship of the α-SMA expression with the other alveologenesis-related processes observed on the cells cultured on the FN-silk membrane. Previous work describing the use of the FN-silk membrane as a substrate for different tissue models^51–54^ has not reported any findings or events suggestive of epithelial-to-mesenchymal transition or stress-related responses of the cells, which, while it cannot be directly extrapolated to the alveolar-capillary model, indicates that the α-SMA expression could be an inherent function of this model. Evaluating, among others, whether α-SMA-positive cells retain an alveolar epithelial phenotype or assume a myofibroblast one, as well as following their fate before and after the alveolar structures mature to fishnet-shaped conformations, would help elucidate the processes driving this novel finding and potentially understand if epithelial cells can assume such functions in vitro in the absence of myofibroblasts. Previous work has shown FN-silk to be an excellent matrix for culturing a range of different primary cells both on the membrane^51,52^ and in different formats^48,49,52^. We thereby hypothesize that an FN-silk-based system would be a highly promising candidate to evaluate barrier formation, surfactant production, and alveologenesis with primary human alveolar cells, providing a better understanding of the tissue’s physiology and function and facilitating personalized medicine applications.

Despite the inherent limitations of H441 cells, our current findings suggest that the FN-silk membrane is an excellent BM mimic, supporting a more physiologically relevant alveolar-epithelial model than its synthetic counterparts in similar setups, thereby providing a potent matrix for modeling additional aspects of alveolar tissue in future work. Due to its strength and elasticity, briefly demonstrated herein and more extensively discussed in previous work^51,55^, the FN-silk membrane could be promising matrix to support dynamic culture conditions that would better recapitulate the alveolar microenvironment. For example, cyclic stretching mimicking the breathing motion could further elucidate the dynamic nature of alveologenesis or shear flow under the basal side of the model would more closely mimic the alveolar capillary environment, allowing for gas-exchange studies. Moreover, incorporation of additional tissue-relevant cell types (e.g., immune or stromal cells) would enable a closer mimicry of the in vivo tissue and expand the potential applications. At a different direction, the FN-silk membrane’s compatibility with high resolution microscopy would allow for a quantification of the alveolar-like 3D conformations (dimensions, number, 3D complexity) and enable a better understanding of the contribution of lung development compounds such as corticosteroids (e.g., dexamethasone). Lastly, our characterization of the alveologenesis-related events observed herein is done largely from a morphological standpoint. However, we believe that future work would benefit from a comparison of the transcriptomic and proteomic profiles of immortalized and primary cell-based models with ex vivo samples of the native tissue, to better elucidate the potential of each model as well as their limitations.

Overall, the FN-silk membrane supports an alveolar-capillary model that mimics key alveolar barrier properties. We herein showed that the developed model mimics important tissue functions such as barrier formation, surfactant and ECM production, and, for the first time in vitro, alveologenesis-related processes. These features make the FN-silk membrane a highly physiologically-relevant alternative to the vastly used synthetic substrates for applications based on immortalized epithelial cells^22,23,27,37,38^. The current work further builds on previous reports^49,51,52,66^ showing that FN-silk is a potent biomaterial for in vitro tissue engineering. In summary, our system provides a promising foundation for more relevant tissue models in respiratory research and regenerative medicine, demonstrating that the FN-silk membrane is a highly favorable substrate for alveolar tissue engineering.

## Methods

### Constructing the alveolar-capillary model

A previously established method using FN-silk’s self-assembly at interfaces was used to prepare the FN-silk membrane^51,52,54–56^. Briefly, the FN-silk protein solution (1.8 mg/ml) (Spiber Technologies, Stockholm, Sweden) was thawed at room temperature, 550 µl/well was placed in a 48-well (Thermo Scientific Nunc, Waltham, MA, USA) and incubated overnight at ambient conditions. The next day, the FN-silk membrane had formed at the liquid-air interface and were captured using a custom- desinged^52,56^ polystyrene insert (Suppl. Fig. 18) to facilitate handling similar to previous setups^51–53,55,56^. The inserts were manufactured by injection molding (Hammarplast Medical AB, Lidköping, Sweden).

On Day 0, H441 cells (2.1 × 10^5^ cells/cm^2^) were seeded on the apical side of the FN-silk membrane and the constructs were cultured submerged for 2 days in a 24-well plate (Thermo Scientific Nunc, Waltham, MA, USA). On Day 2, HPMEC were seeded on the basal side (0.53 × 10^5^ cells/cm^2^) (Fig. 1, Suppl. Fig. 18). Specifically, the inserts were inverted, the endothelial cells were seeded, and were allowed to adhere for 20 min (37^*o*^C, 5% CO_2_, 95% humidity). Thereafter, the inserts were inverted again, placed in a 24-well plate, 200 µl of OptiMEM was added apically, and 1000 µl of MV was added basally. On Day 3, the alveolar-capillary models were moved into differentiation conditions. The epithelial cells were moved to the air-liquid interface, by removing the apical medium and the medium on the basal side (MV) was supplemented with dexamethasone (1 µM) (D4902, Sigma Aldrich, St. Louis, MO, USA). Medium was changed every second day until Day 7 and thereafter 500 µl was changed daily until the final time point, Day 11. Transparent TC inserts for 24 well plates (PET, pore size 0.4 μm) (Sarstedt, Numbrecht, Germany) were treated the same as the FN-silk-based constructs for the TC insert-based cultures.

### Viability and metabolic activity assays

Cell viability was evaluated using Live/Dead Viability/Cytotoxicity Kit (Invitrogen, Waltham, MA, USA). The kit’s reagents were diluted in culture medium (calcein-AM (1:2000) and ethidium homodimer-1 (1:500)) and incubated at (37 °C, 5% CO_2_, 95% humidity) for 30 min. Thereafter, the constructs were imaged directly. The metabolic activity of the cells was monitored at Days 1, 3, 7, and 11 using AlamarBlue™ cell viability reagent (Invitrogen, Waltham, MA, USA). 200 µl of AlamarBlue™ diluted in culture medium (1:10) were added on the apical side and the constructs were incubated at (37 °C, 5% CO_2_, 95% humidity) for 2 h. Two samples (100 µl) per construct were taken from the apical chamber and transferred into a 96-well plate (Greiner Bio-One GmbH, Kremsmünster, Austria). For the sampling conducted on Day 7 and 11 (i.e., when the epithelial cells are under air-lift conditions), the constructs were used only at one timepoint, either on Day 7 (and then discarded), or on Day 11, to avoid any potential effect of rewetting the differentiating epithelial layer. Fluorescence intensity was measured using a CLARIOstar plate reader (BMG LABTECH, Ortenberg, Germany). Mean values were calculated for each condition as follows (*N* = 1): For FN-silk membranes on Day 1 (*n* = 9), Day 3 (*n* = 9), Day 7 (*n* = 3), Day 11 (*n* = 4) and for TC inserts on Day 1 and 3 (*n* = 6), and on Day 7 and 11 (*n* = 3). Values were normalized to the culture area, and fold change relative to Day 1 was calculated for each substrate. Due to the FN-silk membrane thinness and protein composition, additional samples were prepared to compensate for the possibility of accidentally damaging the substrate when handling the constructs during culture. Samples considered potentially damaged during culture were excluded from future measurements.

### Transepithelial electrical resistance (TEER)

Transepithelial electrical resistance (TEER) was measured 3 times per sample using EVOM3 (WPI, Sarasota, FL, USA) combined with the STX2 electrodes (WPI, Sarasota, FL, USA) with the resistance set at 10,000 Ω. TEER was measured both on epithelial-endothelial models (i.e., H441& HPMEC) and on epithelial-only cultures (i.e., H441). Samples were prepared as follows (*N* = 1): for the epithelial-endothelial FN-silk models, measured on Days 1 and 3 (*n* = 10), Day 7 (*n* = 5), and Day 12 (*n* = 4); for epithelial only FN-silk cultures measured on Days 1 and 3 (*n* = 14), Day 7 (*n* = 8), and Day 12 (*n* = 5); for TC inserts, samples were measured on Days 1 and 3 (*n* = 8), and on Days 7 and 12 (*n* = 4 each) for both conditions. TEER was also measured on FN-silk membranes (*n* = 5) and TC-inserts (*n* = 3) without cells. As described above, after Day 3 the constructs were measured once either on Day 7 or on Day 12 to avoid any potential effect of rewetting the differentiating epithelial layer. The final value was calculated by subtracting the mean value of cell-free membranes from that of cell-seeded membranes and was normalized to the growth area of each substrate (Ω cm^2^). To ensure consistency with the overnight (Day 11–12) permeation experiment (described below), TEER was evaluated over 12 days of culture instead of 11.

### Permeation

Fluorescein (376 Da, Sigma Aldrich, St. Louis, MO, USA) and dextran (70 kDa, Invitrogen, Waltham, MA, USA) were used to evaluate passive permeation through the FN-silk and the TC insert membranes with or without cells at the end of culture (Day 11) and overnight (i.e., Day 12). Specifically, the molecules were diluted (fluorescein 3.32 µg/ml and dextran 200 µg/ml) in MV culture medium and 200 µl of the solution was loaded on the apical side of the alveolar-capillary models and on bare FN-silk or TC insert membranes on Day 11. 1000 µl of fresh MV medium were added on the basal side of each construct. 100 µl samples were collected from the basal side the same and following day (i.e., after 1, 4, and 24 h) and replenished with 100 µl of fresh medium. Fluorescence was measured on a Spark 10 M microplate reader (Tecan, Männedorf, Switzerland). Fluorescence was converted to concentration using a standard curve and the values were corrected for sampling. The percentage of permeation was calculated compared to the positive control. Mean values were calculated for each condition as follows (*N* = 1): FN-silk membrane with cells (*n* = 4) or without cells (*n* = 5), and TC insert with cells (*n* = 5) or without cells (*n* = 3). Statistical testing was performed using a two-way ANOVA with Tukey’s multiple comparisons performed in GraphPad Prism (6.01).

## Supplementary Information

Below is the link to the electronic supplementary material.

Supplementary Material 1

## Data Availability

Data are available as supplementary materials published together with this article.
