# Supplementary material for: FN-silk membrane enables alveologenesis processes and self-organization of the H441 epithelial cell line into native-like alveolar morphology
Source: Sci Rep. 2026 Jun 27;16:19634. doi: 10.1038/s41598-026-59951-4 (PMC13310257; doi:10.1038/s41598-026-59951-4)
Supplement: Supplementary file 1 — Supplementary Material 1 [file 41598_2026_59951_MOESM1_ESM.docx]

**Supplementary data**

**FN-silk membrane enables alveologenesis processes and self-organization of H441 epithelial cell line into native-like alveolar morphology**

Savvini Gkouma^1^, Linnea Påvenius^2^, Linnea Gustafsson^3^*^,^*^4^, Christos Tasiopoulos^1^, André Charbonneau^7^, Swapna Upadhyay^5^, Hjalmmar Brismar^6^, Lena Palmberg^5^, and My Hedhammar^1^*^,∗^*

1Division of Protein Technology, Department of Protein Science, KTH Royal Institute of Technology, Stockholm, Sweden

2Science for Life Laboratory, Department Women’s and Children’s Health, Karolinska Institute, Stockholm, Stockholm, Sweden

3Division of Micro and Nanosystems, Department of Intelligent Systems, School of Electrical Engineering and Computer

Science, KTH Royal Institute of Technology, Stockholm, Sweden

4Spiber Technologies AB, Stockholm, Sweden

5Institute of Environmental Medicine, Integrative Toxicology, Karolinska Institute, Stockholm, Sweden

6Science for Life Laboratory, Department of Applied Physics, KTH Royal Institute of Technology, Stockholm, Sweden

7Atlas Antibodies, Stockholm, Sweden

*∗*[myh@kth.se](mailto:myh@kth.se)

1. **Supplementary methods**

**Cell culture**

NCI-H441 (H441) immortalized cells isolated from the pericardial fluid of a patient with papillary adenocarcinoma of the lung (ATCC, VA, US) and human pulmonary microvascular endothelial cells (HPMEC) (PromoCell, Heidelberg, Germany) were expanded in T75 flasks (Sarstedt, Nümbrecht, Germany). H441 cells were used between passage 31-36 and cultured in OptiMEM medium (Gibco, Waltham, MA, USA) supplemented with 10% heat inactivated fetal bovine serum (FBS) and 1% penicilin and streptomycin. HPMEC were used between passage 3-5 and cultured in MV endothelial cell growth medium (PromoCell, Heidelberg, Germany) complete with MV supplement mix (PromoCell, Heidelberg, Germany) and supplemented with 1% penicilin and streptomycin. Medium was changed every second day and cells were enzymatically harvested with TrypLE (Thermo Fisher Scientific, Waltham, MA, USA) upon reaching 80% confluence.

**Sample fixation and immunostaining**

***Whole mount samples***

The models developed on either the FN-silk or the TC insert membranes intended for whole mount fluorescence stainings were fixed as described in previous work^51–53,56^, specifically by incubating them in 4% paraformaldehyde (PFA) for 10 minutes. They were thereafter washed twice in PBS and stained as described below. Samples intended for different analyses (i.e., paraffin embedding and sectioning, TEM, and SEM) were fixed as described in the respective sections.

***Fluorescence stainings***: Samples were permeabilized in 0.1% Triton-X in PBS for 10 minutes, and washed in 0.2% Tween in PBS (PBST) for an additional 10 minutes. They were thereafter blocked in goat serum (Sigma-Aldrich, St. Louis, MO, USA) for 1 hour before adding the respective primary antibody as summarized in Suppl. table 1 either apically or basally. After overnight incubation at +4^o^C the samples were washed for 15 minutes in PBST and the secondary antibody (Suppl. table 1) was added either apically or basally. The incubation with the secondary antibody was performed for 2 hours in darkness at room temperature. Finally, the nuclei were counterstained with DAPI. Samples including F-actin stainings were incubated in phalloidin for 30 minutes before the nuclear staining (Suppl. table1).

In addition to antibody stainings, wheat germ agglutinin (wga) was used to label glycoproteins. Briefly, the samples were incubated in wga for 10 minutes before permeabilization and the previously described staining steps were followed thereafter.

***Histology***

In preparation for the Atlaplex staining, FN-silk based samples were processed according to the manufacturers instructions. Specifically, the samples were fixed for 10 minutes in 37% formaldehyde and washed twice in PBS. After fixation, the samples were incubated in 70% ethanol (EtOH) for 3 days to facilitate membrane dehydration and unmounting from the insert. The ethanol-dehydrated membranes were manually unmounted from the inserts and embedded in 1% agarose blocks prior to dehydration and paraffin embedding. The agarose embedded samples were dehydrated with an automated system (Miles, Tissue-Tek V.I.P. E150/E300 Series). Embedding was performed at 40 ^o^C using the following sequence: 70% EtOH (30 & 60 min), 95% EtOH (45, 45, 60 min), 99% EtOH (45, 60, 60 min), xylene (60, 80 min), paraffin (60, 60, 120 min). Thereafter the samples were subjected to three changes of paraffin (two 1 hour long and one 2 hour long) at 60 ^o^C. Extracted from the automated handling machine, the rigid blocks from the dehydrated agarose were cut in half and —the interface revealed by the cut— was placed so that it was first exposed to the microtome’s blade. 10 µm thick cross sections were collected.

**Table 1. List of antibodies (Ab) and other stains**

| **Primary Ab** | **Species** | **Clonality** | **Dilution** | **Manufacturer** |
| --- | --- | --- | --- | --- |
| ZO-1 | mouse anti-human | monoclonal (clone: ZO1-1A12) | 1:200 | Invitrogen (33-9100) |
| Ki-67 | rabbit anti-human | monoclonal (clone: EPR3610) | 1:500 | Abcam (92742) |
| CD31 | mouse anti-human | monoclonal (clone: MEM-05) | 1:200 | Acris GmbH (BM4047) |
| VE-cad | mouse anti-human | monoclonal (clone: BV9) | 1:1000 | Invitrogen (MA1-198) |
| pro-SPC | rabbit anti-human | polyclonal | 1:1000 | Abcam (ab90716) |
| PDPN | rabbit anti-human | polyclonal | 1:500 | Human protein atlas (HPA007534) |
| alpha ENaC | rabbit anti-human | polyclonal | 1:500 | Thermo Fisher Scientific (PA1-920A) |
| *α*-SMA | rabbit anti-human | polyclonal | 1:100 | Thermo Fisher Scientific (PA5-19465) |
| Cav-1 | rabbit anti-human | polyclonal | 1:500 | Abcam (ab2910) |
| COL4A2 | rabbit anti-human | polyclonal | 1:100 | Human protein atlas (HPA069337) |
| LAMA1 | mouse anti-human | polyclonal | 1:200 | Abcam (ab11575) |
| LAMA5 | rabbit anti-human | polyclonal | 1:100 | Human protein atlas (HPA058389) |
| **Secondary Ab** | **Species** | **Clonality** | **Dilution** | **Manufacturer** |
| AlexaFluor 488 | goat anti-mouse | polyclonal | 1:2000 | Invitrogen (A21121) |
| AlexaFluor 488 | goat anti-rabbit | polyclonal | 1:2000 | Invitrogen (A11034) |

| **Conjucated labels** | **Dilution** | **Manufacturer** |
| --- | --- | --- |
| Alexa Fluor 488 Phalloidin | 1:400 | Invitrogen (A12379) |
| Alexa Fluor 594 Phalloidin | 1:400 | Invitrogen (A12381) |
| Wheat Germ Agglutinin (WGA), Rhodamine | 1:1000 | Vector Laboratories (RL-1022) |

**Atlaplex staining**

The manufacture’s protocol for kit AP-S3 (Atlas Antibodies, Stockholm, Sweden) was followed. Briefly, specific biotin conjugated antibodies were incubated with streptavidin-horseradish peroxidase (HRP). Then, excess streptavidin was inactivated by excess biotin. The samples were incubated with antibodies (Suppl. table [2](#_bookmark7)) bearing biotin and streptavidin-HRP either for 60 min at 20 ^o^C or overnight at 4 ^o^C. After each individual antibody incubation, the fluorophore was enzymatically processed for 25 min, then inactivated. For every antibody, the same cycle was repeated.

**Table 2. List of antibodies (Ab) and fluorophores**

| **Primary Ab** | **Species** | **Clonality** | **Concentration (***µ***g/***µ***l)** | **Manufacturer** |
| --- | --- | --- | --- | --- |
| SFTPB | rabbit anti-human | polyclonal | 0.000056 | Atlas Antibodies (HPA062148) |
| COL4A2 | rabbit anti-human | polyclonal | 0.0015 | Atlas Antibodies (HPA069337) |
| PECAM1 | mouse anti-human | monoclonal (CL14693) | 0.000050 | Atlas Antibodies (Amab91986) |

| **Fluorophores** | **Dilution** |
| --- | --- |
| Tyramide 555 | 1:25 |
| Tyramide 594 | 1:25 |
| Tyramide 647 | 1:25 |

**Microscopy**

***Widefield Microscopy***

Whole mount constructs were imaged using a non-automated inverted fluorescence microscope (Nikon Eclipse Ti) unless otherwise stated. The entire culture area was inspected and an overview (i.e., 2x or 4x) image of the entire construct was captured when possible, depending on the marker of interest. Thereafter, representative areas of each construct were imaged. Depending on the marker of interest different (e.g., three or more) areas of the construct are imaged, and a representative area was selected. In cases where the marker of interest was not homogeneously located throughout the culture area, more than one representative image was selected instead.

***Confocal Airyscan Microscopy***

For the characterization of cellular morphology and protein expression, whole mount FN-silk-based alveolar capillary models were fixed on Day 11 and prepared as previously described.

**Image Acquisition**

High-resolution images were acquired using an inverted Zeiss LSM 980 confocal microscope equipped with an Airyscan 2 detector (Carl Zeiss Microscopy GmbH, Jena, Germany). Image acquisition was done in Airyscan CO-8Y mode. To minimize spectral bleed-through, fluorophores (DAPI, Alexa Fluor 488, Alexa Fluor 594) were imaged sequentially.

Detailed morphological analysis and protein localization (ENaC and *α*-SMA) was performed using a 40X/1.2 C-Apochromat Water immersion objective. The specific imaging parameters for these high-magnification volumes were: i) for small volumes (Figure [5](#_bookmark4)): A field of view of 162.19 x 162.52 x 29.07 *µ*m (1968 x 1972 x 171 pixels) composed of 171 Z-planes, and ii) for large volumes (Figure [6](#_bookmark5)): A field of view of 162.19 x 162.52 x 49.47 *µ*m (1968 x 1972 x 292 pixels) composed of 292 Z-planes. For large-scale overview and assessment of the entire construct (Figure [6](#_bookmark5) b.i,i’,i”), a 10X/0.3 EC-Plan NeoFluar objective was utilized. This overview volume was generated from a tile scan of 9 x 9 (81) tiles in 30 Z-planes, resulting in total dimensions

of 5.34 x 5.36 x 0.088 mm (16192 x 16250 x 30 pixels).

**Image Processing and Analysis**

All raw Airyscan data were processed using ZEN Blue software (version 3.12, Carl Zeiss Microscopy GmbH, Jena, Germany). Post-acquisition processing included Airyscan 2D/3D reconstruction and the generation of 3D projections, maximum intensity projections, and orthogonal slices to visualize the lumen formation and cellular organization within the 3D model.

***Multiplex Acquisition of Histology Sections Using Laser Confocal Microscope***

Leica’s SP8 microscope equipped with tunable white light laser and a separate 405 diode was used to excite the fluorophores. Excitation matched the fluorophore excitation peaks. Emission wavelengths were successfully separated by implementing near emission peaks 10-30 nm detector thresholds and gating when necessary. Acquisition was performed sequentially and the detector was of type HyD and in standard mode. Laser power never exceeded 10%.

***SEM and FIB***

The samples with cells (N=1, n=3 for both FN-silk and TC-inserts) fixed in 2% glutaraldehyde in 0.1 M HEPES buffer and left at +4 °C overnight similar to previous work^51,53^. The following day, the samples were washed 3 times with 0.1 M HEPES buffer for 5 minutes each. The samples were then serially dehydrated in 50, 70, 96% ethanol for 10 minutes, two times each, and 100% ethanol for 15 minutes, three times each. Samples were then chemically dried in 2 parts 100% ethanol and 1 part hexamethyldisilazane (HMDS, Sigma Aldrich, St. Louis, MO, USA) for 15 minutes, 1 part 100% ethanol and 1 part HMDS for 15 minutes, 1 part 100% ethanol and 2 parts HMDS for 15 minutes and finally, 3 times in HMDS alone for 15 minutes each. The last HMDS was evaporated overnight and the samples were coated with a 12 nm thick layer of gold through metal evaporation, and then imaged using SEM (Gemini Ultra 55, Zeiss, Oberkochen, Germany).

The samples without cells (N=1, n=2) were serially dehydrated in the same way, gold coated using a table-top sample sputter (MCM-100, SEC) and imaged and etched using a FIB/SEM (Helios 5 UC, FEI, USA). The ion etching was done for 3 seconds using 30 kV, and the images were acquired using the SE mode at 10 kV.

***TEM***

Cells on FN-silk membranes (N=1, n=2) were fixed in 2.5% glutaraldehyde in 0.1M phosphate buffer, pH 7.4 at room temperature for 1 hour similar to previous work^53^. After fixation silk membranes were rinsed in 0.1M phosphate buffer pH 7.4 prior to post-fixation in 2% osmium tetroxide in 0.1M phosphate buffer, pH 7.4 at 4°C for 2 hours. The samples were then stepwise dehydrated in ethanol, followed by acetone, and finally flat embedded in LX-112. Transversal ultra thin sections ( 60–80 nm) of the samples were prepared using an EM UC7 ultra-microtome (Leica, Wetzlar, Germany) and contrasted with uranyl acetate followed by Reynolds´s lead citrate. TEM imaging was performed in a Hitachi HT7700 transmission electron microscope (Hitachi High-Technologies, Tokyo, Japan) operated at 80 kV and digital images were acquired using a Veleta CCD camera (Olympus Soft Imaging Solutions, Münster, Germany).

**FN-silk membrane bulging**

The flexibility of the FN-silk membrane was visualized by trapping an air column below the membrane and increasing the hydrostatic pressure with a surrounding water column. In short, the insert with the FN-silk membrane was turned upside down, positioned on a hollow cylinder, and placed inside a large beaker. Water was poured into the beaker, capturing air inside the cylinder which pressurized the membrane. Photographs were taken with a standard camera (5d mark iii, Canon, Japan).

1. **Supplementary figures**


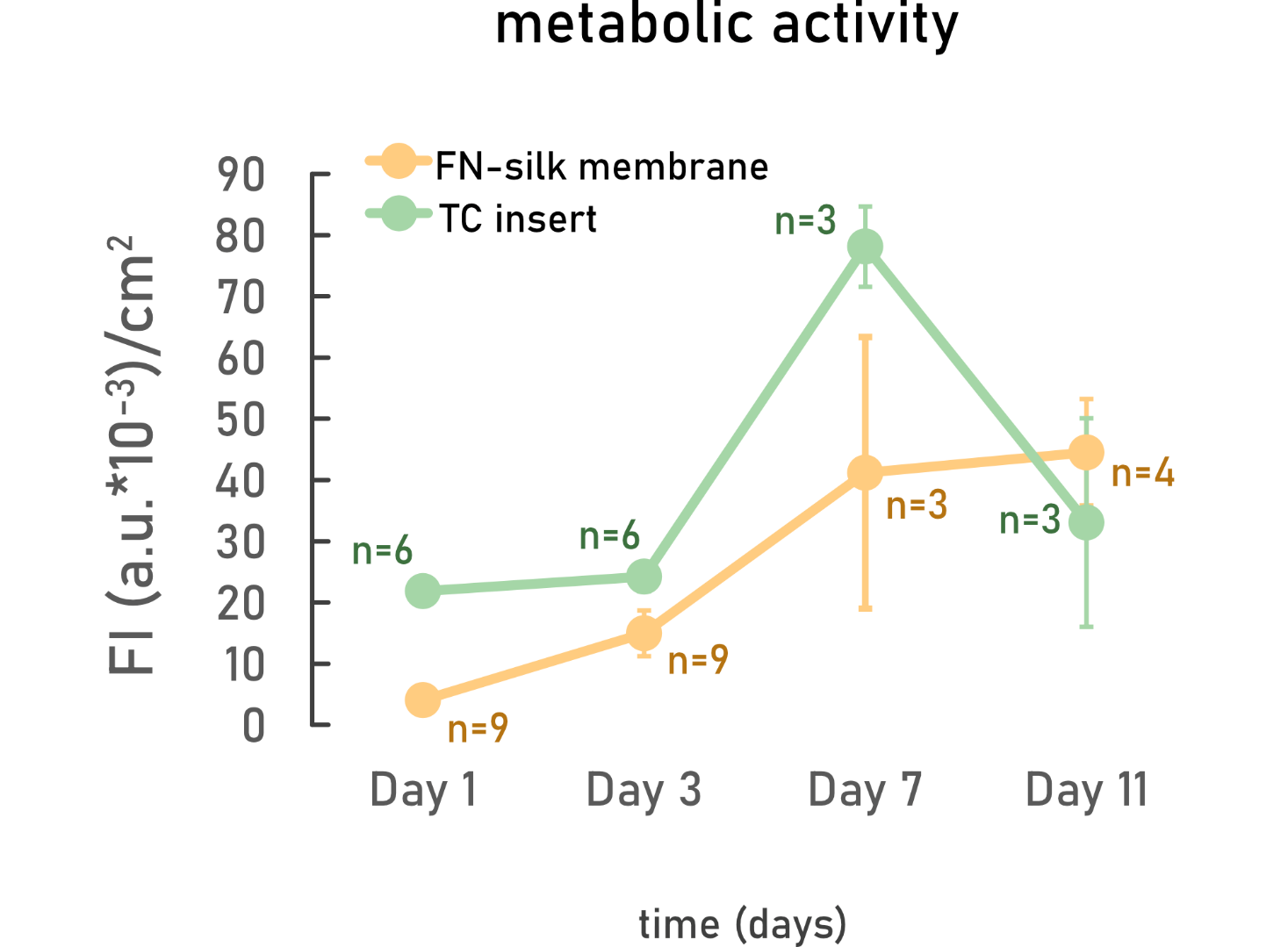


**Suppl. Figure** [**1**](#_bookmark8). Cell metabolic activity measured with AlamarBlue*^TM^* assay throughout the culture period of the alveolar-capillary model cultured on the FN-silk membrane or TC insert, reported as fluorescence intensity (FI) normalized by the culture area of each substrate. (n*≥*3) (mean ± SD).


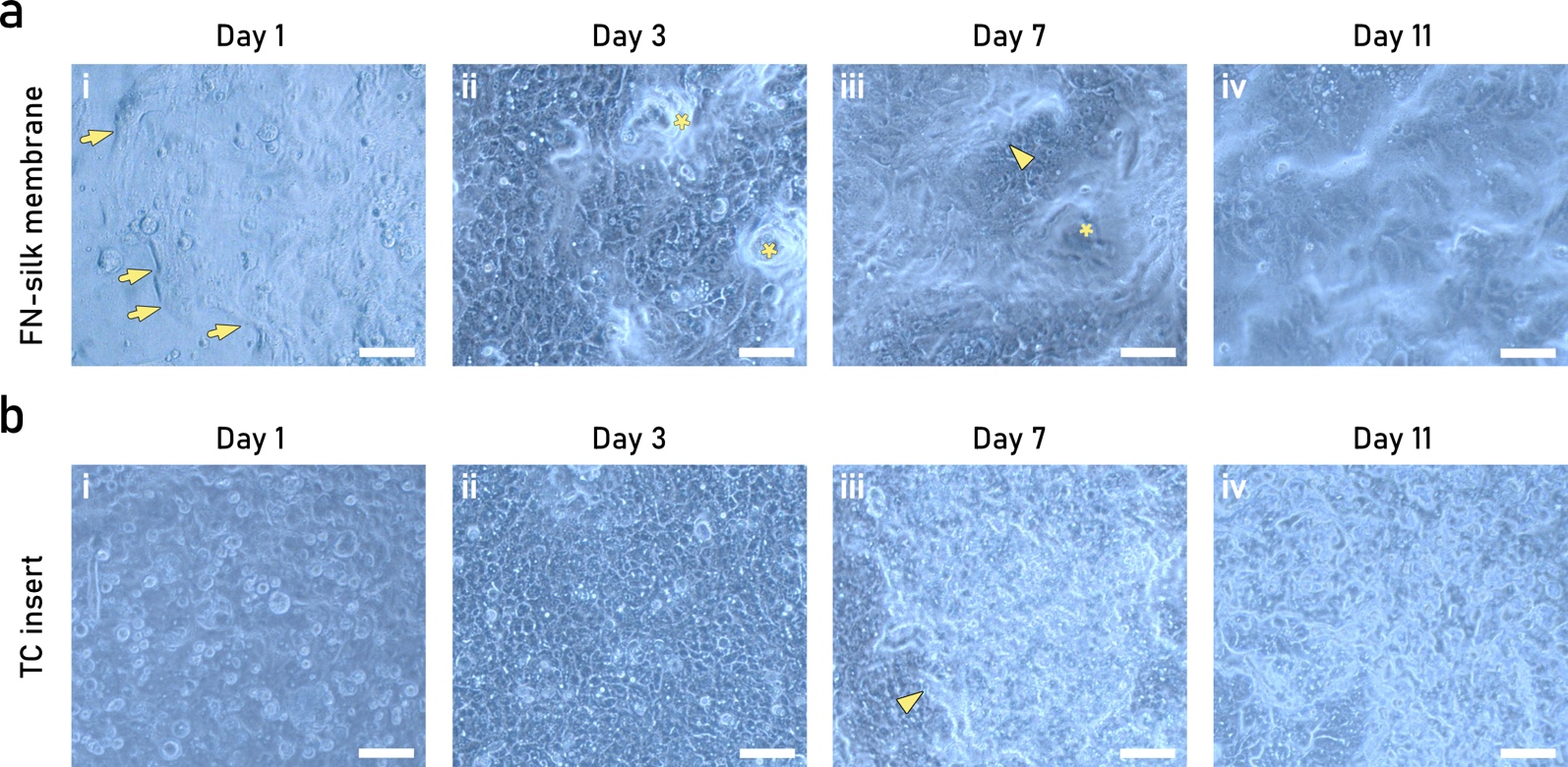


**Suppl. Figure** [**2**](#_bookmark1). Brightfield imaging of the epithelial layer of ongoing cultures on the FN-silk membrane **(a)** or the TC insert **(b)**, at different time points (i.e., Days 1, 3, 7, and 11). On Day 1, cells appeared to be fewer in number and more spread on the FN-silk membrane **(a.i)** compared to the TC insert counterpart **(b.i)**. Parts of the FN-silk membrane were still not covered by cells (arrows indicate the cell layer-membrane border). By Day 3 **(a.ii, b.ii)**, both cultures were confluent. However, cells cultured on FN-silk were larger in size and showed early signs of stratification (asterisks) **(a.ii)**. Cells on the TC inserts on the other hand, were smaller in size and arranged as a tightly packed monolayer without any signs of stratification. By Day 7 **(a.iii, b.iii)** the stratification of the FN-silk-based cultures continued (**a.iii**), while the basal layer was still visible (arrowhead). The TC-based cultures **(b.iii**), showed no signs of stratification. Rather, large areas of cell aggregates covered most of the culture area. The basal monolayer was still visible (arrowhead) while the cell size had decreased compared to Day 3. The same trend continued until Day 11 with the stratified layers expanding on the entire culture area of the FN-silk membrane **(a.iv)** and the cell aggregates covering most of the TC insert culture area **(b.iv)**. Scale bars 100 *µ*m.


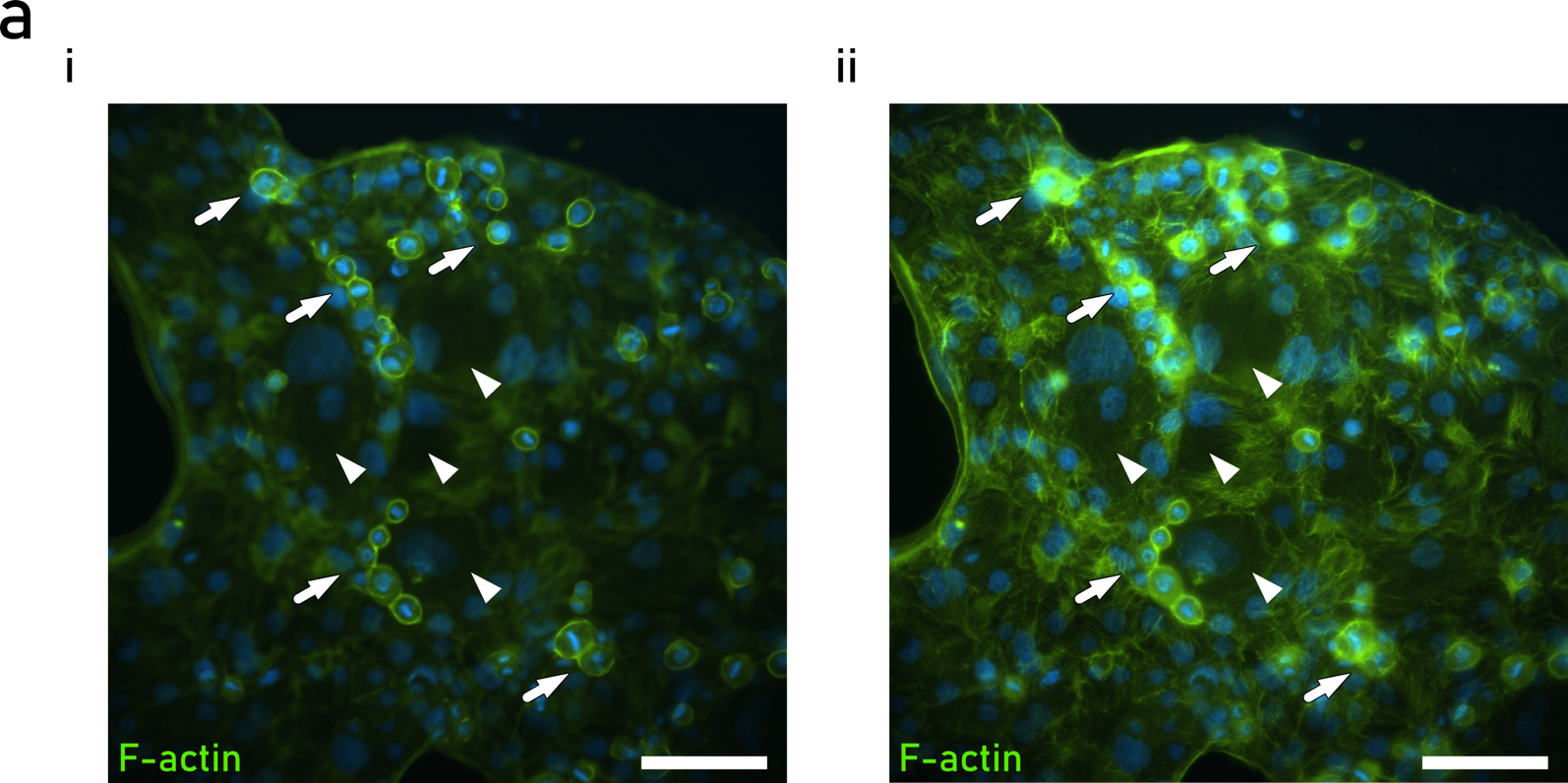


**Suppl. Figure 3**. **Immunofluorescence imaging showing cell morphology at early time points. a)** FN-silk-based culture on Day 2. The same culture area is imaged in different focal points bringing in focus **a.i:** tall, columnar cells, resembling the ATII morphology (arrows) or **a.ii:** large, flattened cells, resembling the ATI morphology (arrowheads). F-actin filaments are stained green. Nuclei are counterstained with DAPI (blue). Scale bars: 100 *µ*m.


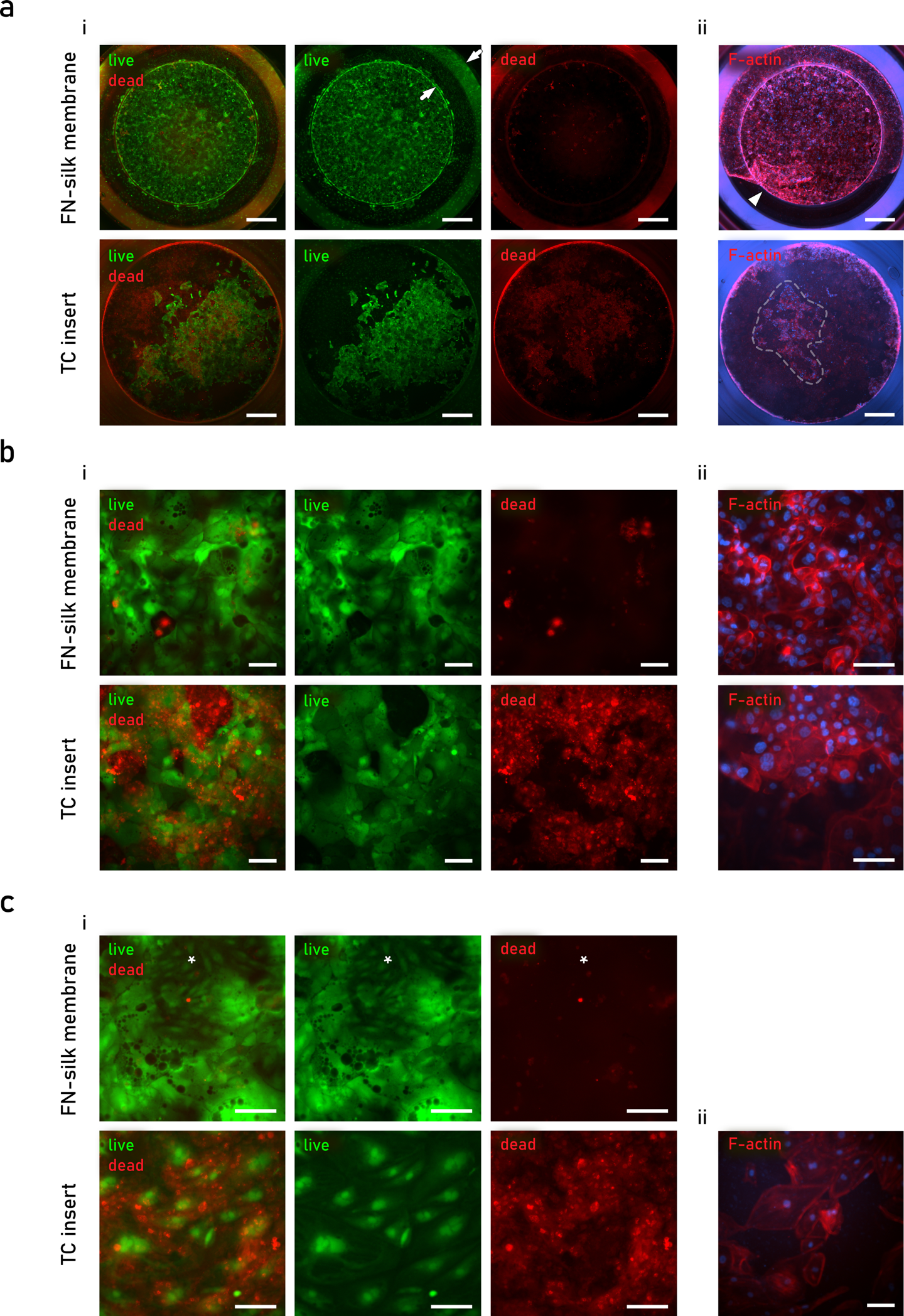


**Suppl. Figure** [**4**](#_bookmark3)**. FN-silk- (top) or TC insert-based (bottom) alveolar-capillary models after prolonged culture (Day 19).** Immunofluorescence imaging of viable (green) and dead (red) cells (a.i, b.i, c.i) (N=1, n=1) and cell morphology (F-actin, red) (a.ii, b.ii, c.iii) (N=1, n=1) . **a)** Overview of the entire culture area. **Top:** Arrows indicate the pipette rest, (area further described in Suppl. Figure [18](#_bookmark20), accessible only to endothelial cells covered by viable endothelial cells (a.i). The same area was damaged during imaging resulting in FN-silk membrane detaching from the insert and folding (arrowhead) (**a.ii**). **Bottom:** A small number of epithelial cells remained on the TC insert, located on the border of the culture area and the marked area (dashed line). Zoomed in details showing epithelial (**b**) and endothelial (**c**) cell viability (i) and morphology (ii) on both substrates. **c**) Due to the presence of a fully viable epithelial monolayer on FN-silk cultures, the endothelial cells can only be visualized in specific areas and distinguished from the epithelial ones due to their different shape and signal intensity (asterisk). In contrast, large areas of the TC insert cultures lack viable epithelial cells making imaging of the viable endothelial cells simpler. F-actin filaments are stained (phalloidin, red) and nuclei counterstained with DAPI (blue) (a-c.ii). Scale bars: 1000 *µ*m (a), 100 *µ*m (b,c).


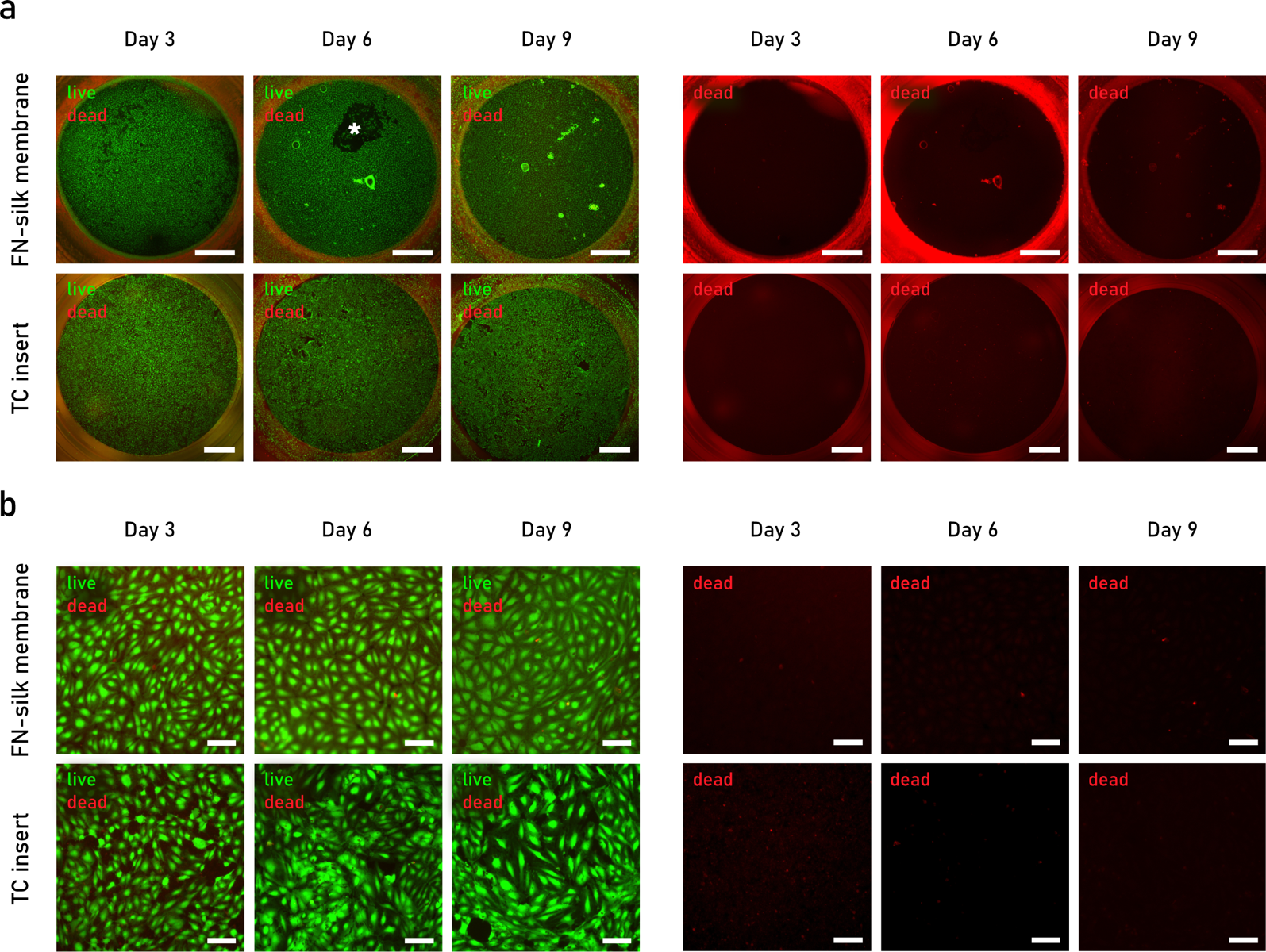


**Suppl. Figure** [**5**](#_bookmark4). **Immunofluorescence stainings of HPMEC monocultures on the FN-silk membrane (top) or the TC insert (bottom).** The viability of the endothelial cells when cultured on the basal side of either substrate is evaluated at different time points (i.e,. Day 3, 6, 9). Live cells are stained green and red cells red (N=1, n=1) . **a)** Overview of the entire culture area, **b)** zoomed in detail. Manual scraping during imaging created an artifact (asterisk) (a). Scale bars: 1000 *µ*m (a), 100 *µ*m (b).


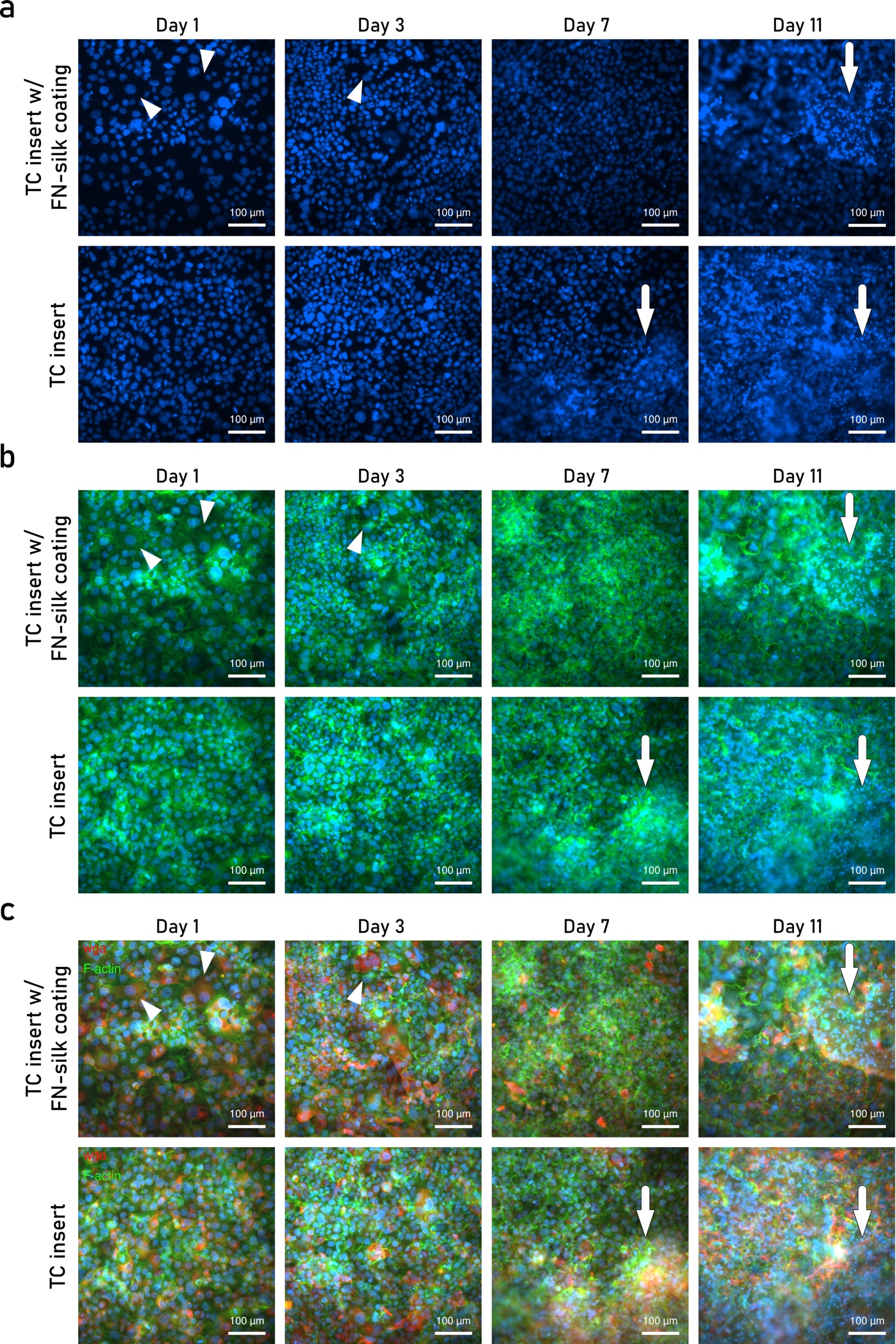


**Suppl. Figure** [**6**](#_bookmark5). Immunofluorescence images of epithelial cell (H441) monocultures at different time points (i.e., Days 1, 3, 7, 11) based on TC inserts with (top) or without (bottom) an FN-silk coating. Cells with a flattened morphology (arrowheads) and dead cell aggregates (arrows) are indicated. F-actin filaments (phalloidin, green) (b, c) and glycoproteins (wga, red) (c) are stained. Nuclei are counterstained with DAPI (blue). Scale bars: 100 *µ*m.


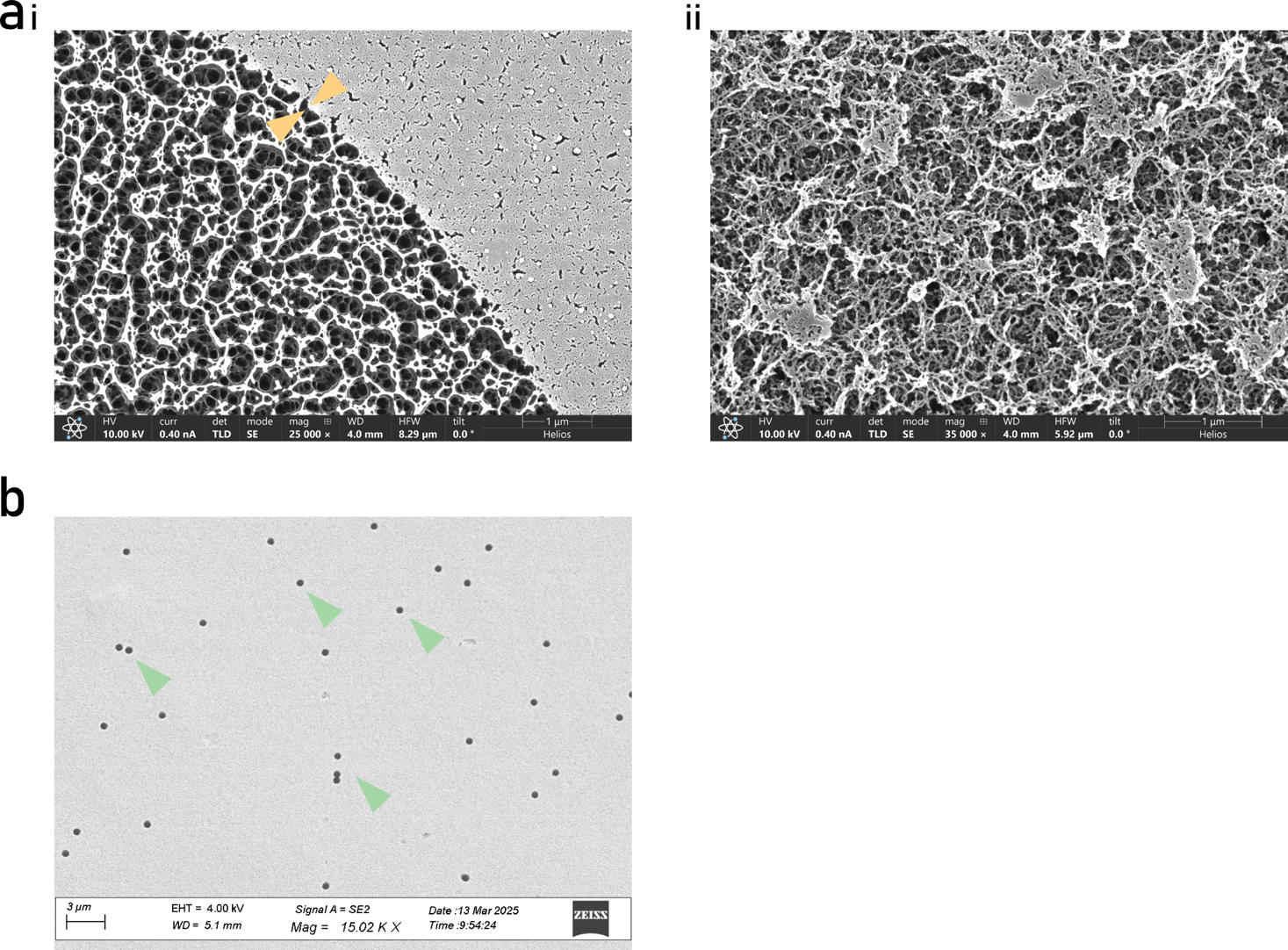


**Suppl. Figure** [**7**](#_bookmark9). SEM images of the FN-silk membrane (a) and the TC insert (b) without cells. The apical side of the FN-silk membrane has a smooth texture on the air-exposed side (a.i). This thin smooth layer is followed by a mesh of interconnected cavities visualized here after FIB ion etching (a.i). The interface between the etched and non-etched membrane is marked (arrowheads). The interconnected mesh is also visible on the basal side of the FN-silk membrane (a.ii) which appears textured and lacking the smooth layer of the apical side. b) The TC insert is smooth and features track etched (0.4 *µ*m) pores marked with arrowheads.


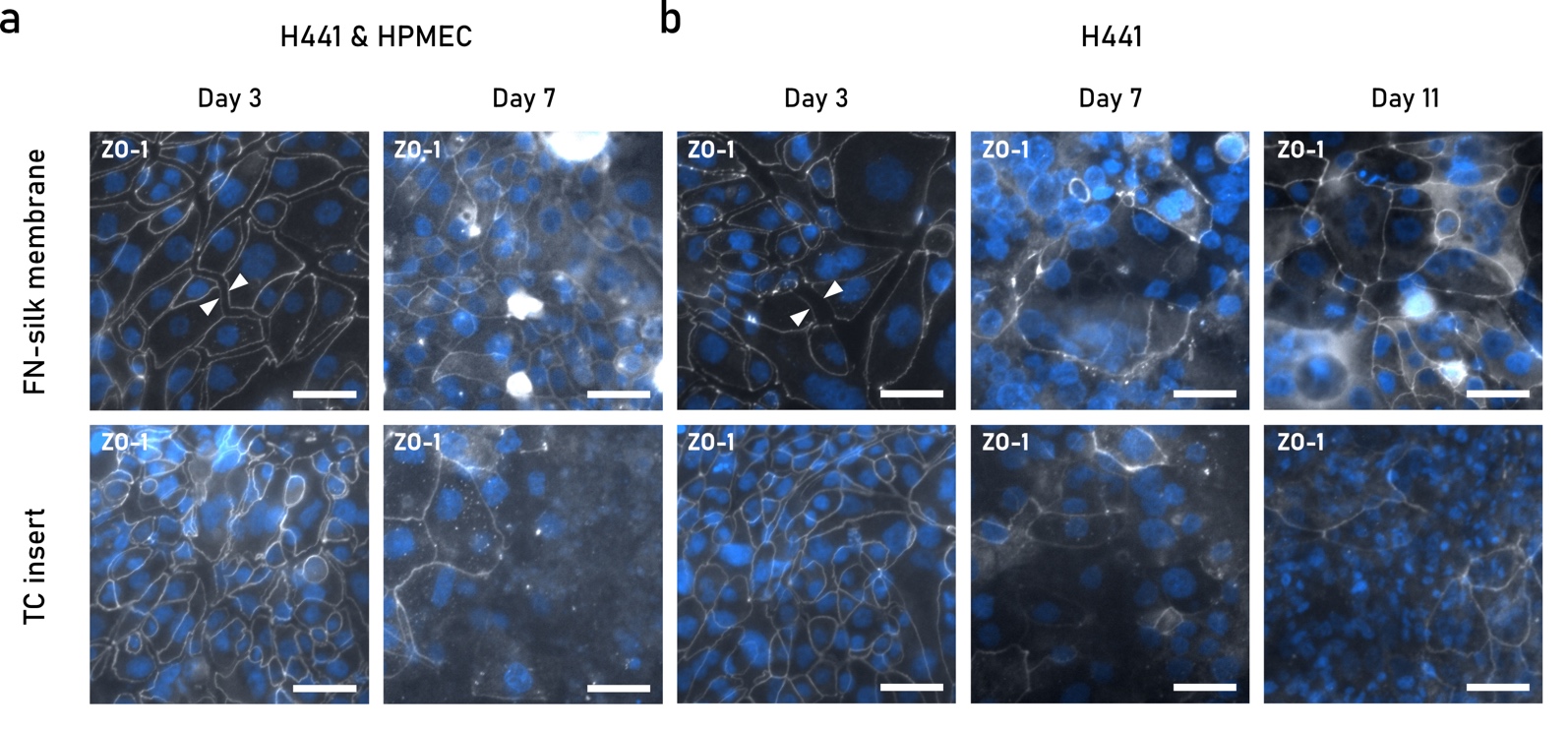


**Suppl. Figure 8**. Immunofluorescence imaging of tight junction formation on FN-silk or TC insert cultures at different time points (not shown in Figure 3c). **a)** Co-cultures (i.e., H441 and HPMEC) on Day 3 and Day 7 (N=1, n=1) . **b)** Monocultures (i.e., H441 only) at Days 3, 7, 11 (N=1, n=1). Arrowheads indicate areas of a loose tight junction network. ZO-1 (white), DAPI (blue). Scale bars: 50 *µ*m.


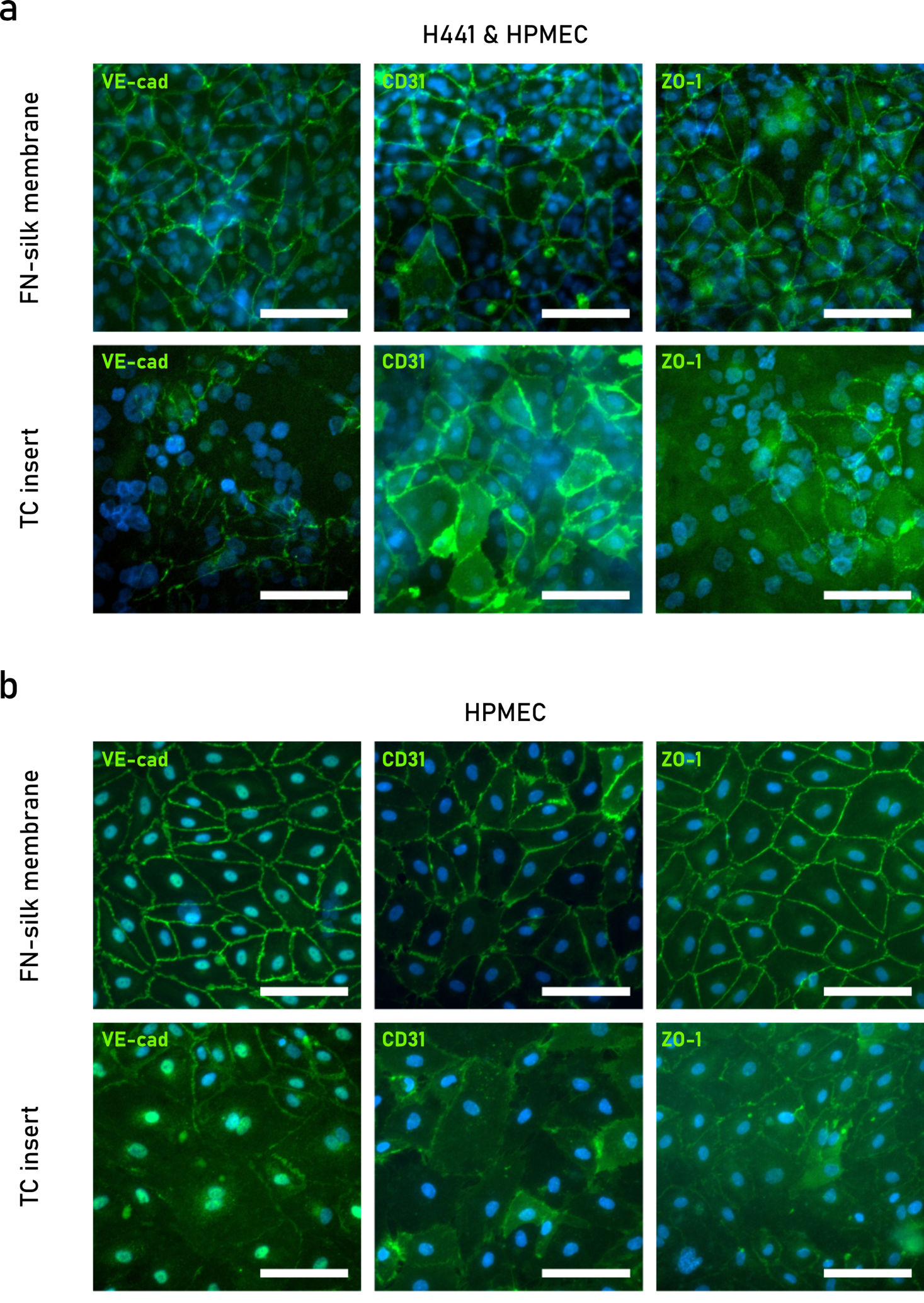


**Suppl. Figure 9**. Immunofluorescence imaging of endothelial cells on Day 11, cultured as part of the alveolar-capillary model (i.e., co-cultures) (N=1, n=1) (a) or as single seeded cultures (HPMEC monocultures) (N=1, n=1) (b) on either the FN-silk (top) or the TC insert (bottom) membranes. Adherence junctions (VE-cad, green), endothelial marker CD31 (green), or tight junctions (ZO-1, green) are stained. Nuclei are counterstained with DAPI (blue). Scale bars: 100 *µ*m.


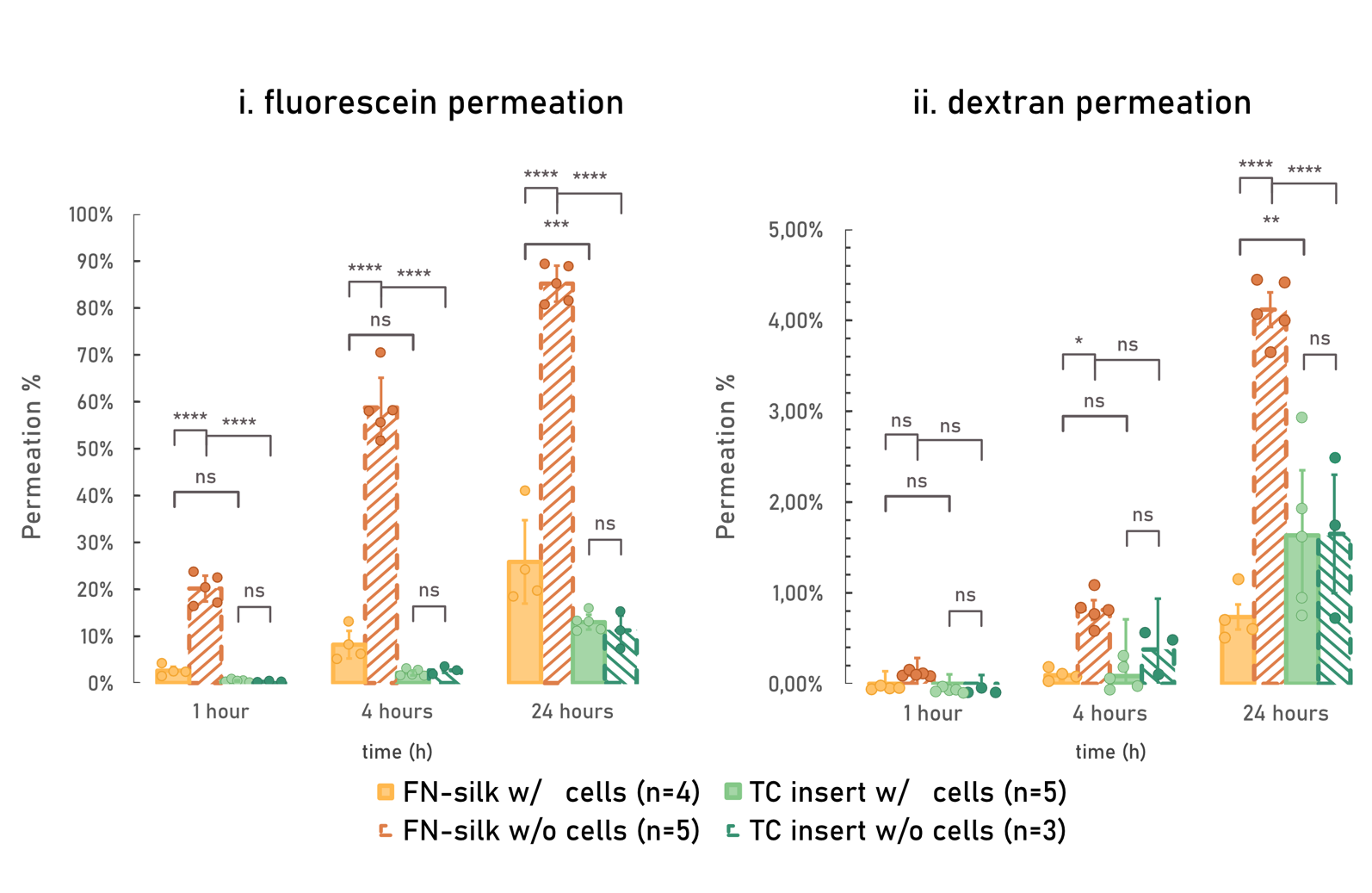


**Suppl. Figure** [**10**](#_bookmark12). Permeation (mean ± SD) of fluorescein (i) and 70 kDa Dextran (ii) through FN-silk or TC insert-based alveolar-capillary models at Day 11 and overnight (Day 1112). Samples (n*≥*3) were collected after 1, 4, and 24 hours. For the statistical analysis, a two-way ANOVA with Tukey’s multiple comparisons was performed. ns: non-significant, *: p *≤* 0.05, **: p *≤* 0.01, ***: p *≤* 0.001, ****: p *≤* 0.0001.


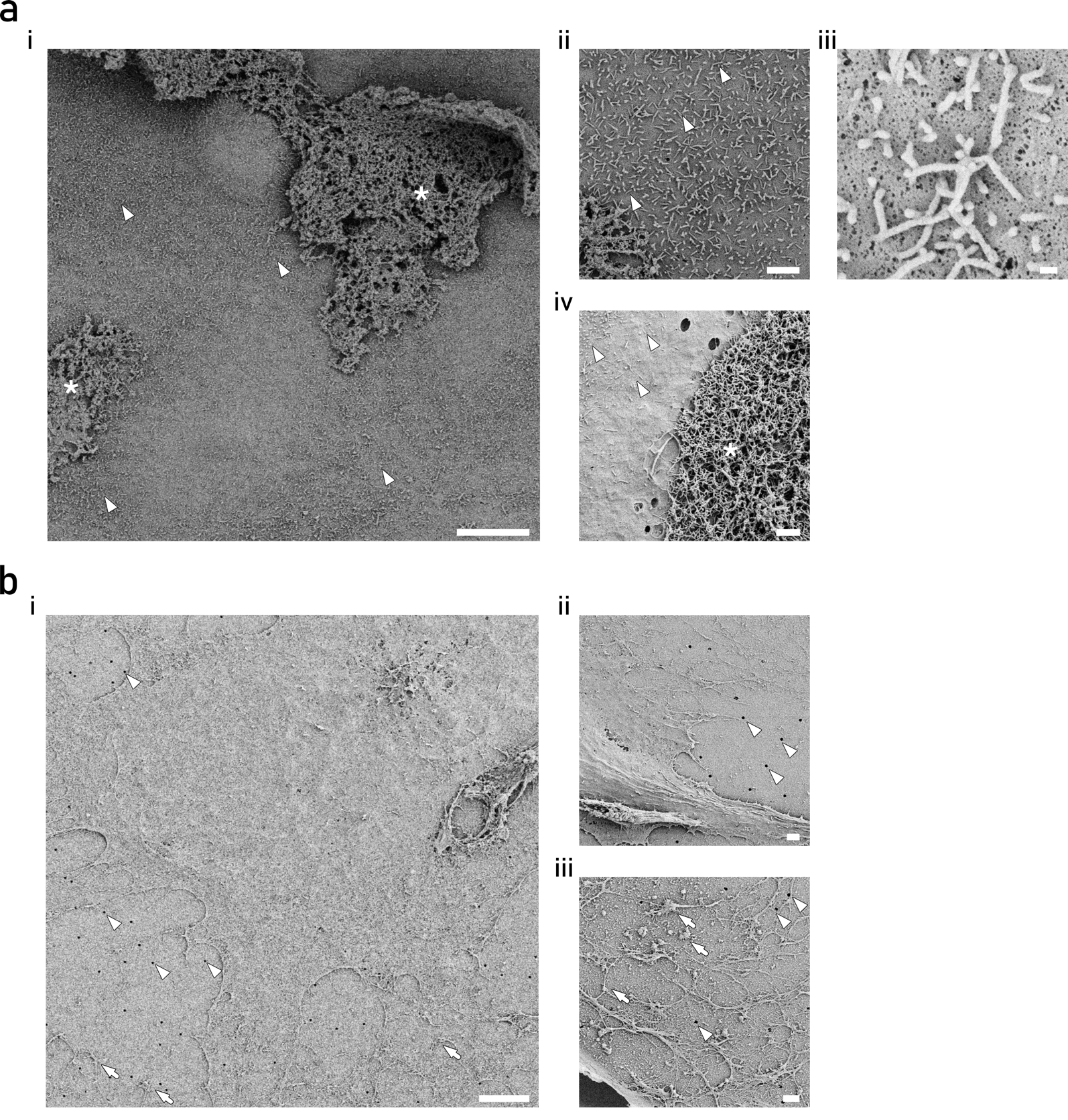


**Suppl. Figure** [**11**](#_bookmark13). SEM imaging of the apical side of co-cultures (i.e., H441 and HPMEC) on Day 11. **a)** Top view of the FN-silk-based models. i) Overview, ii) microvilli-rich cells, iii) zoomed-in detail, and iv) extracellular secretion-rich area (asterisk). Examples of microvilli (arrowheads) and secretion-rich areas (asterisks) are marked. **b)** Top view of the TC based models. The cell layer is sparse making the pores of the PET membrane visible (arrowheads). i) Overview, ii) zoomed-in detail, iii) shrunk cells with rounded morphology are visible (arrows) Scale bars: 10 *µ*m (a.i, b.i), 2 *µ*m (a.ii, a.iv, b.ii-iii) and 200 nm (a.iii).


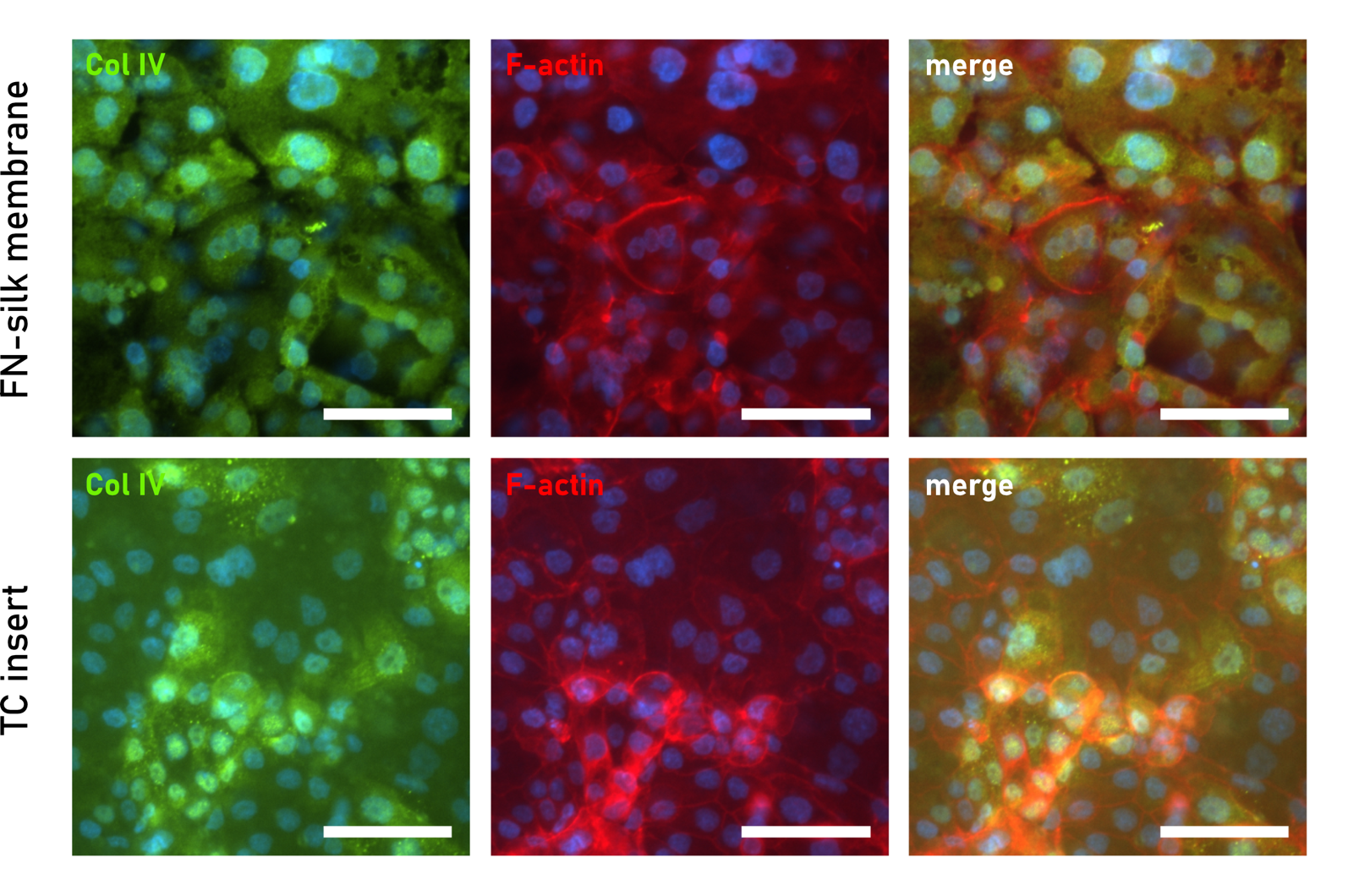


**Suppl. Figure** [**12**](#_bookmark14). Immunofluorescence images of epithelial cells of the alveolar-capillary models on the FN-silk membrane or the TC insert at Day 11, stained for col IV (green) and F-actin (phalloidin, red), (N=1, n=1). Nuclei are counterstained with DAPI (blue). Scale bars: 100 *µ*m.


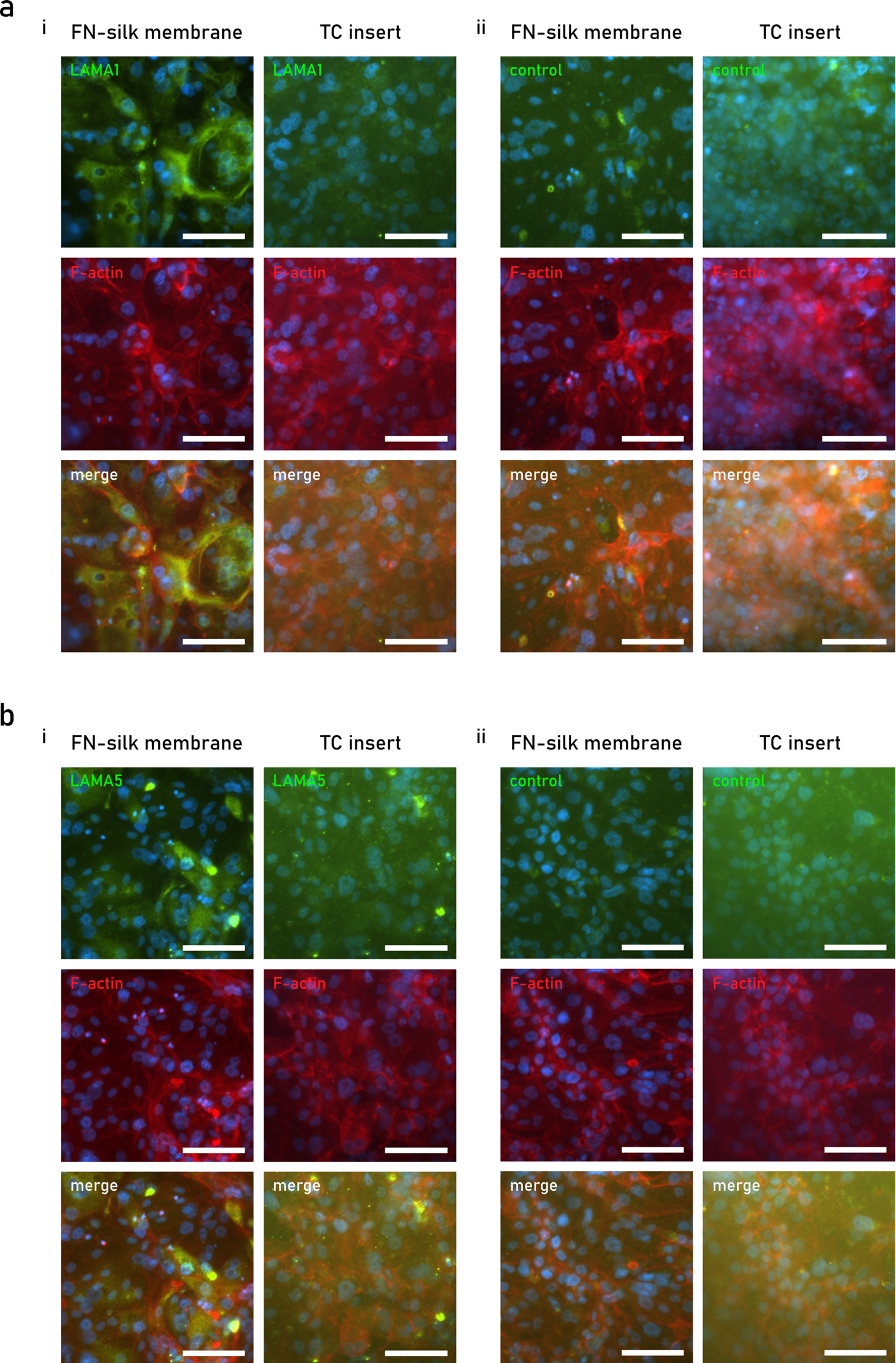


**Suppl. Figure** [**13**](#_bookmark15). Immunofluorescence images of the epithelial cells (Day 11) of the alveolar-capillary models based on the FN-silk membrane or the TC insert, stained for LAMA1 (green) (N=1, n=1) (a.i) or LAMA5 (green) (N=1, n=1) (b.i) and F-actin (phalloidin, red). In the control stainings where the primary antibody is excluded (a.ii, b.ii) only background autofluorescence is detected in the green channel (TC inserts). Nuclei are counterstained with DAPI (blue). Scale bars: 100 *µ*m.


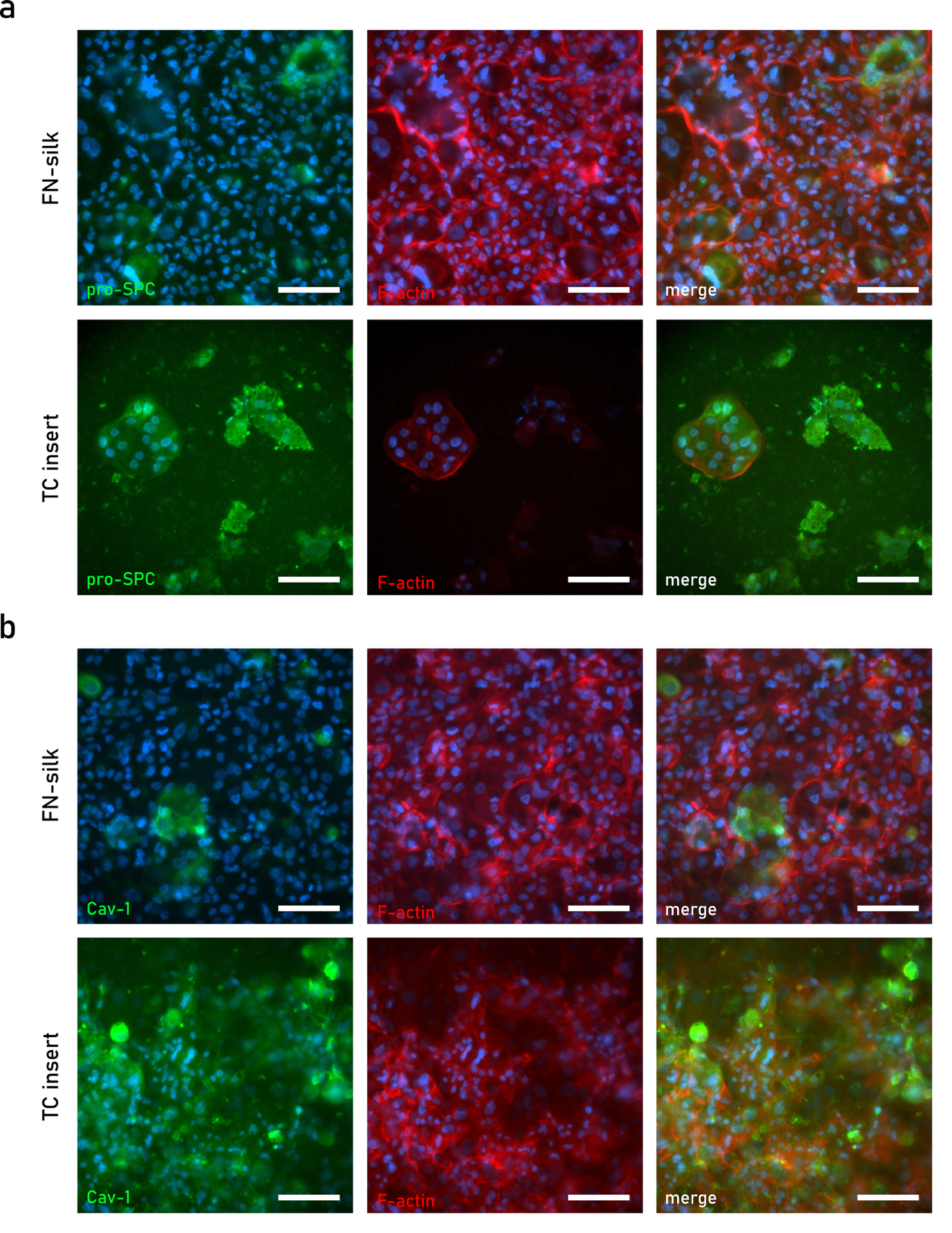


**Suppl. Figure** [**14**](#_bookmark16). Immunofluorescence images of single seeded FN-silk membranes or TC inserts (H441 monocultures) at Day 11, stained for pro-SPC (green) (FN-silk: N=4, n=1, TC-insert: N=1, n=1) (a) or Cav-1 (FN-silk: N=2, n=1, TC=insert: N=1, n=1) (b) and F-actin (phalloidin, red). FN-silk (top), TC insert (bottom). Nuclei are counterstained with DAPI (blue). Scale bars: 100 *µ*m.


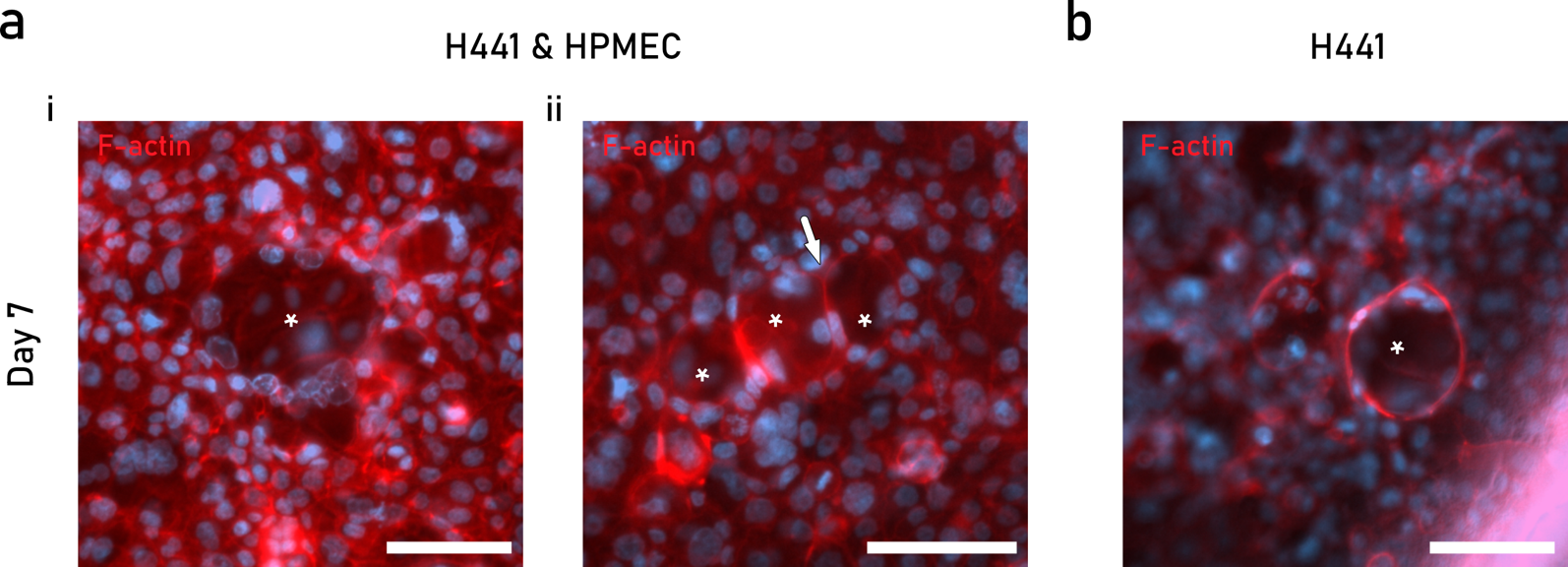


**Suppl. Figure** [**15**](#_bookmark17). Saccular alveolar conformations detected on co-cultures (H441 and HPMEC) (a) and single seeded (H441 monocultures) (b) on FN-silk-based cultures at Day 7. Septation (arrow) and formation of more complicated structures can be detected on the co-cultures (a.ii). Lumens are indicated (asterisk). F-actin is stained (phalloidin, red) and nuclei are counterstained with DAPI (blue). Scale bars 100 *µ*m.


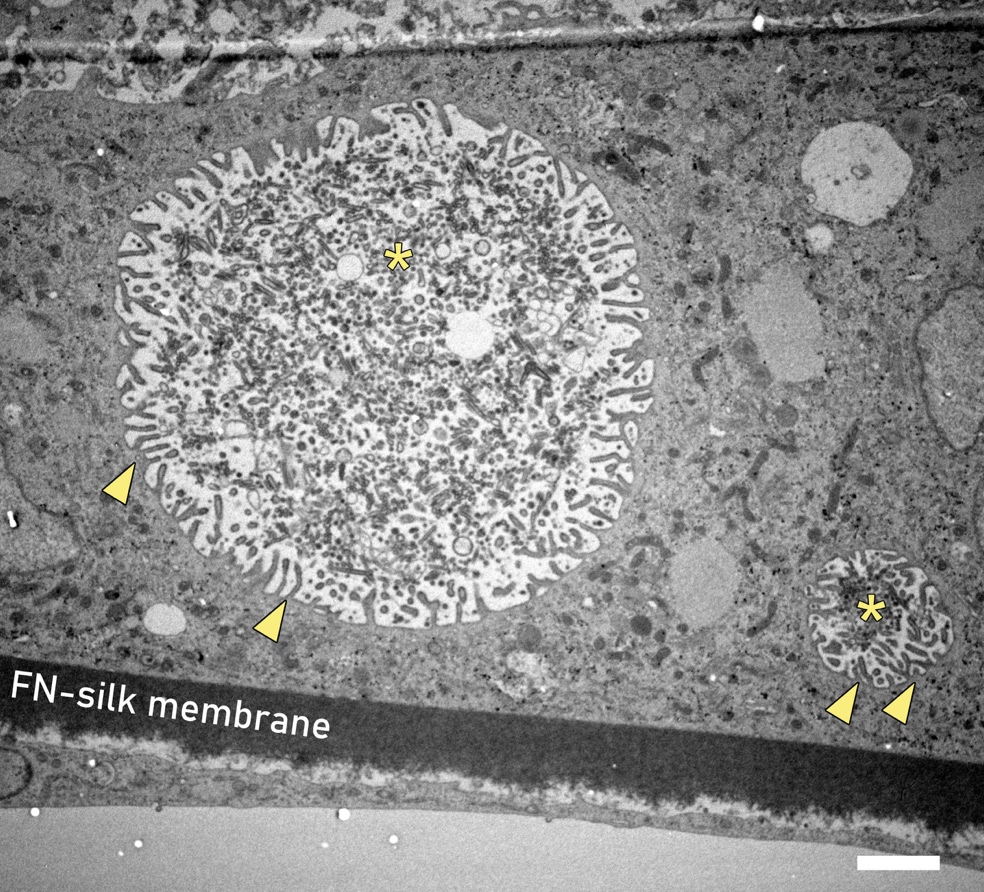


**Suppl. Figure** **[16](#_bookmark18)**. TEM image of a cross section (XZ) of the alveolar-capillary FN-silk-based model on Day 11. Alveolar lumens (asterisks) are enclosed by microvilli (arrowheads)-bearing epithelial cells. Cell secretions are visible inside the lumens. An endothelial cell is visible on the basal side of the FN-silk membrane. Scale bar: 2 *µ*m.


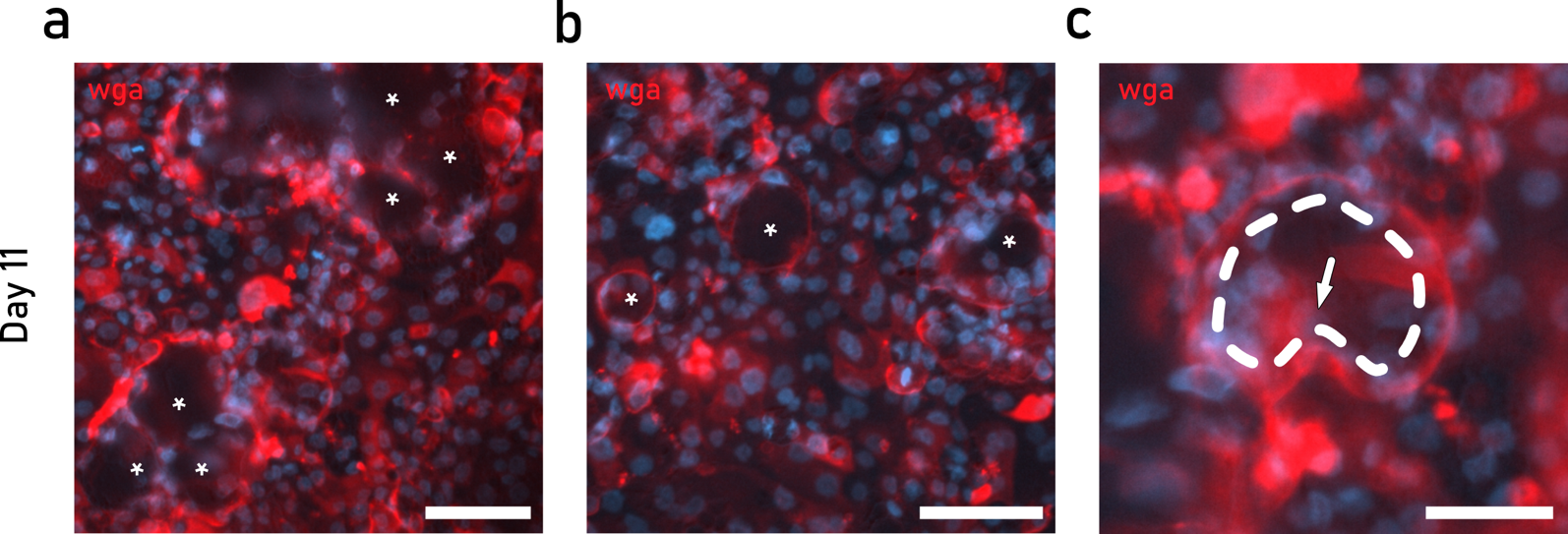


**Suppl. Figure** [**17**](#_bookmark19). Alveologenesis detected on single seeded (H441 monocultures) FN-silk-based cultures at Day 11. **a)** Hollowing and later stage interconnected alveolar conformations, **b)** saccular alveolar conformations, and **c)** septation (arrow), can be detected. Lumens (asterisk) and typical septa *ω*-shape (dashed line) are indicated. Cells are stained for wga (red) and nuclei are counterstained with DAPI (blue). Scale bars: 100 *µ*m (a,b), 50 *µ*m (c).


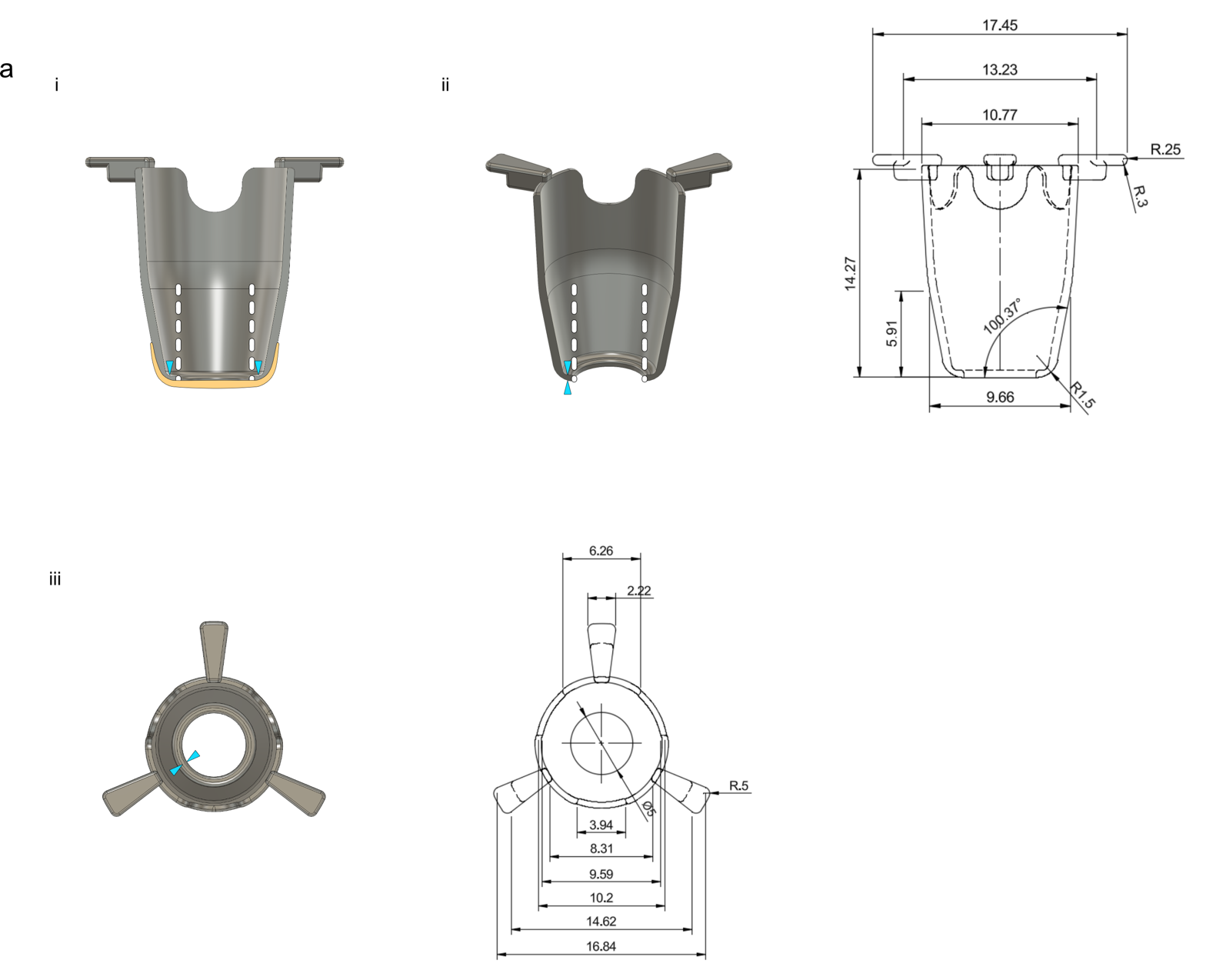


**Suppl. Figure** [**18**](#_bookmark20). Schematic illustrations of the inserts used to capture the FN-silk membrane (in yellow (i)) seen as: cross sections (i, ii) and top view (iii). The FN-silk membrane adheres on the bottom side of the insert wrapping around it as indicated

1. The apical side culture area is marked (i, ii) (dashed lines) and is visible in the top view (iii) as a white colored area. To facilitate medium exchange without touching the FN-silk membrane a pipette-rest ring was implemented close to the culture area marked with green arrowheads (i-iii). Representative dimensions (mm) of the design are shown.
